# DNA methylation regulates discrimination of enhancers from promoters through a H3K4me1-H3K4me3 seesaw mechanism

**DOI:** 10.1186/s12864-017-4353-7

**Published:** 2017-12-12

**Authors:** Ali Sharifi-Zarchi, Daniela Gerovska, Kenjiro Adachi, Mehdi Totonchi, Hamid Pezeshk, Ryan J. Taft, Hans R. Schöler, Hamidreza Chitsaz, Mehdi Sadeghi, Hossein Baharvand, Marcos J. Araúzo-Bravo

**Affiliations:** 1 Department of Bioinformatics, Institute of Biochemistry and Biophysics, University of Tehran, Tehran, Iran; 2 Computer Science Department, Colorado State University, Fort Collins, CO USA; 3 Department of Stem Cells and Developmental Biology, Cell Science Research Center, Royan Institute for Stem Cell Biology and Technology, ACECR, Tehran, Iran; 4 Department of Computer Engineering, Sharif University of Technology, Tehran, Iran; 5 Computational Biology and Systems Biomedicine, Biodonostia Health Research Institute, 20014 San Sebastián, Spain; 6 Department of Cell and Developmental Biology, Max Planck Institute for Molecular Biomedicine, Münster, Germany; 7 School of Mathematics, Statistics and Computer Science, College of Science, University of Tehran, Tehran, Iran; 8 School of Biological Sciences, Institute for Research in Fundamental Sciences (IPM), Tehran, Iran; 9 Illumina Inc., San Diego, USA; 10 Medical Faculty, University of Münster, Münster, Germany; 11 National Institute of Genetic Engineering and Biotechnology (NIGEB), Tehran, Iran; 12 Department of Developmental Biology, University of Science and Culture, Tehran, Iran; 13 Computational Biology and Bioinformatics Group, Max Planck Institute for Molecular Biomedicine, Münster, Germany; 14 IKERBASQUE, Basque Foundation for Science, 48011 Bilbao, Spain

**Keywords:** DNA methylation, Histone modifications, Promoters, Enhancers, H3K4me1, H3K4me3, Computational epigenomics, Next generation sequencing

## Abstract

**Background:**

DNA methylation at promoters is largely correlated with inhibition of gene expression. However, the role of DNA methylation at enhancers is not fully understood, although a crosstalk with chromatin marks is expected. Actually, there exist contradictory reports about positive and negative correlations between DNA methylation and H3K4me1, a chromatin hallmark of enhancers.

**Results:**

We investigated the relationship between DNA methylation and active chromatin marks through genome-wide correlations, and found anti-correlation between H3K4me1 and H3K4me3 enrichment at low and intermediate DNA methylation *loci*. We hypothesized “seesaw” dynamics between H3K4me1 and H3K4me3 in the low and intermediate DNA methylation range, in which DNA methylation discriminates between enhancers and promoters, marked by H3K4me1 and H3K4me3, respectively. Low methylated regions are H3K4me3 enriched, while those with intermediate DNA methylation levels are progressively H3K4me1 enriched. Additionally, the enrichment of H3K27ac, distinguishing active from primed enhancers, follows a plateau in the lower range of the intermediate DNA methylation level, corresponding to active enhancers, and decreases linearly in the higher range of the intermediate DNA methylation. Thus, the decrease of the DNA methylation switches smoothly the state of the enhancers from a primed to an active state. We summarize these observations into a rule of thumb of one-out-of-three methylation marks: “In each genomic region only one out of these three methylation marks {DNA methylation, H3K4me1, H3K4me3} is high. If it is the DNA methylation, the region is inactive. If it is H3K4me1, the region is an enhancer, and if it is H3K4me3, the region is a promoter”. To test our model, we used available genome-wide datasets of H3K4 methyltransferases knockouts. Our analysis suggests that CXXC proteins, as readers of non-methylated CpGs would regulate the “seesaw” mechanism that focuses H3K4me3 to unmethylated sites, while being repulsed from H3K4me1 decorated enhancers and CpG island shores.

**Conclusions:**

Our results show that DNA methylation discriminates promoters from enhancers through H3K4me1-H3K4me3 seesaw mechanism, and suggest its possible function in the inheritance of chromatin marks after cell division. Our analyses suggest aberrant formation of promoter-like regions and ectopic transcription of hypomethylated regions of DNA. Such mechanism process can have important implications in biological process in where it has been reported abnormal DNA methylation status such as cancer and aging.

**Electronic supplementary material:**

The online version of this article (10.1186/s12864-017-4353-7) contains supplementary material, which is available to authorized users.

## Background

Multicellular organisms need to establish tissue- and temporal-specific transcriptional programs from a single genome sequence. Such programs coordinate transcription factors (TFs), chromatin-remodeling, chromatin-modifying enzymes, DNA methylation and DNA functional elements such as promoters, insulators, and enhancers. In a previous study on the interaction between DNA methylation and TFs, we found that the methylation-resistant CpG methylation motifs (CpGMMs) are in crosstalk with TFs in gene expression regulation [1]. Such crosstalk could be explained by two mechanisms. One, proposed by Schübeler’s group [2], according to which the TFs binding to DNA regions protect them from being methylated. Another mechanism [1] might be that the methylation-resistant CpGMMs signal the TFs to recruit DNA sequence-specific unmethylation machinery. The two mechanisms are not exclusive and might apply cooperatively. Enhancers, making up 10% of the human genome [3, 4] are the most abundant class of regulatory elements. They up-regulate transcription independently of their orientation or distance to the Transcription Start Sites (TSSs), which makes the comprehensive identification of enhancers more difficult than that of other regulatory elements such as promoters (characterized by 5′-sequencing of genes), or insulators (generally bound by the CCCTC-binding factor, CTCF).

Since the first reports on the presence of methyl groups on some genomic cytosines, huge effort has been made to decrypt the function of DNA methylation, focused mostly on promoters, CpG islands and gene bodies, whereas open questions remain about the role of DNA methylation in enhancers [5]. Additionally, DNA methylation has a determinant role in regulating cell fate at distal regulatory regions rather than promoters and gene bodies [6]. Thus, a better understanding of DNA methylation depletion over enhancers is a crucial, yet cumbersome task due to the genomic and epigenomic complexities of the eukaryotic genomic structure.

Some chromatin modifications are employed in addition to the DNA sequence for a more accurate discrimination between promoters and enhancers [7]. Enhancers and promoters can be distinguished by the methylation status at H3K4. Enhancers are enriched for monomethylation of the 4th lysine of histone 3 (H3K4me1) [8], whereas high levels of trimethylation (H3K4me3) predominantly mark active or poised promoters [9]. However, H3K4me1 alone is not a definitive predictor of enhancer [10–12]. Additional chromatin features at enhancers specify three subcategories of enhancers: (i) Active enhancers: They have activation marks (H3K4me1 and H3K27ac), are bound by the Mediator complex [13], and exert regulatory function to increase the transcription of target genes and produce RNA. (ii) Primed enhancers: Enhancers can exist in a primed state prior to activation, they are marked with activation histone modifications (H3K4me1), which do not yield RNA. (iii) Poised enhancers: They are similar to primed enhancers, but distinguished by the presence of the repression mark (H3K27me3), which must be removed for the transition to an active enhancer state [9, 14].

Most of the genome-wide DNA methylation and histone modification studies on mammalian cells show inverse correlation between DNA methylation and histone H3K4 methylation [15–22]. Specifically, DNA methylation is associated with the absence of H3K4 methylation (H3K4me0) [23]. The interaction between DNA and histone methylation is regulated by a cross-talk in the cell between DNA Methyl-Transferases (DMTs) that can contain domains recognizing methylated histones and Histone Methyl-Transferases (HMTs) containing domains recognizing non-methylated DNA. These interactions involve DNA Methyl-CpG-Binding Domains (MBDs) recognizing DNA methylated CpGs, and zinc finger CXXC domains recognizing non-methylated DNA. Thus, several mechanisms based on the interaction between protein-H3K4me recognizing domains (ADD) [24] and protein-DNA methylation recognizing domains (CXXC and MBD) have been discovered that explain the cross-talk between H3K4 (mono-, di- and tri-) methylation and DNA methylation:

(i) The DMTs activity is regulated by the chromatin-interacting ATRX-DNMT3-DNMT3L (ADD) domain of Dnmt3a that recognizes H3K4me specifically. The ADD domain binds to the histone H3 tail that is unmethylated at lysine 4 [25, 26] and the chromatin methylation activity of Dnmt3a and Dnmt3a/3l is guided by interaction of the ADD domain with the histone H3 tail [27].
(ii) In mammals, there are six lysine-specific HMTs, of the COMPASS (COMplex of Proteins Associated with Set1) MLL/SET1 family, namely four Mixed Lineage Leukemia (MLL1 through 4) and two SET domain containing proteins (SET1A and SET1B) [9]. The MLL1/2 contain a CXXC domain, and use it to recognize DNA unmethylated CpG-rich regions [28–30] whereas the MLL3/4 lack the CXXC domain [31–33]. SET1B and SET1B also lack the CXXC domain. They make a complex with the CXXC finger protein 1 (CFP1), and use the CXXC domain of CFP1 to recognize DNA unmethylated CpG-rich regions [34–38]. CFP1 organizes genome-wide H3K4me3 in embryonic stem cells (ESCs) [39]. Although MLL/SET1 family proteins contain similar HMT catalytic SET domains and are capable of mono-, di-, and tri-methylation of H3K4, the transition from mono- to higher methylation states requires additional subunits [40]. Specifically, the robust tri-methylation activity appears to be mediated by the accessory subunit of the tryptophan-aspartic acid (WD) repeat domain 82 (WDR82) protein that binds SET1A/B but not the MLL proteins [41, 42].
  The CXXC domain of CFP1 allows preferential binding of CFP1 to H3K4me3 at promoters. In contrast, other HMTs, such as the Trr/MLL3/MLL4 complex, lacking the CXXC domain [31–33], are likely responsible for deposition of H3K4me1 at enhancers [9]. This enhancer-promoter discrimination can be explained by differences in DNA sequence, with high number of CpG islands (usually hypomethylated) observed at most promoters, but not at enhancers [43].
(iii) Alongside with these zinc finger CXXC domain recognizing non-methylated DNA is the MBD family of proteins recognizing DNA methylated CpGs. The MBD domain of the MBD1 protein binds more efficiently to methylated DNA within a specific sequence context, and a functional MBD domain is necessary and sufficient for recruitment of MBD1 to these *loci*, while DNA binding by the CXXC domain is largely dispensable [44].

While the use of ChIP-seq improved our knowledge of enhancer chromatin states, many questions related to chromatin state and enhancer function remain unanswered, such as the prediction and functional validation of putative enhancers, the determination of the genes associated with enhancers on a large scale, the disclosing of the mechanism that maintains histone marks at enhancers, the determination of whether poised enhancers contact their target promoters, and the defining of the direction of the flow of influence between the enhancer chromatin state and the target DNA promoter state: whether histone marks define enhancers, or histone marks are rather a consequence of the establishment of the enhancer state [3].

To find the interplay of DNA methylation with other epigenetic marks, we integrated high throughput profiles of DNA methylation, histone modification, DNA binding proteins and gene transcription in several mouse cell types (Table 1). After estimating the correlation of DNA methylation with different histone marks within different DNA regulatory regions, we demonstrated that H3K4me1 has different deposition from other active chromatin marks in regard to DNA methylation. We compared the impact of the 5-methylcytosine (5mC) and 5-hydroxymethylcytosine (5hmC) forms of DNA methylation on the regulation of H3K4 methylation, and uncovered their biological consequences. Most importantly, we developed a hypothesis that explains the role of DNA methylation in regulating a seesaw mechanism between H3K4me1 and H3K4me3, and provided additional evidence for the existence of such mechanism by integrating high throughput datasets of functional analyses obtained from gene knockout (KO) experiments.

**Table 1:** High throughput profiles of DNA methylation, histone modification, transcription and protein binding data sets analyzed in this study. ID represents accession identifiers of the datasets in the National Center for Biotechnology Information (NCBI) Gene Expression Omnibus (GEO) [45, 92] or the European Molecular Biology Laboratory - European Bioinformatics Institute (EMBL-EBI) ArrayExpress [73, 93] databases, along with the reference publications. Target marks are the types of molecular targets represented in the datasets. Cell type notation: mouse embryonic stem cell (ESC), mouse embryonic fibroblast (MEF), wild type (WT), knockout (KO)*.* Sequencing methods notation: Whole-Genome Bisulfite Sequencing (WGBS), Tet-Associated Bisulfite sequencing (TAB-seq), Reduced Representation Bisulfite Sequencing (RRBS), Chromatin Immunoprecipitation sequencing (ChIP-seq), RNA sequencing (RNA-seq)

Row	GEO ID	Target marks	Cell type/tissue	Method	Reference
1	GSE30206	DNA methylation	ESC	WGBS	Stadler et al. [56]
2	GSE44760	DNA methylation	MEF (WT, *Dnmt1* KO)	RRBS	Reddington et al. [59]
3	GSE36173	DNA hydroxymethylation	ESC	TAB-seq	Yu et al. [79]
4	GSE29218	H3K4me1, H3K4me3, Pol2, CTCF, H3K27ac, P300	ESC, MEF, Cortex, Liver	ChIP-seq	Shen et al. [45]
5	GSE12241	H3, H4K20me3, H3K36me3, H3K9me3	ESC, MEF	ChIP-seq	Mikkelsen et al. [38]
6	GSE28254	H3K27me3	ESC	ChIP-seq	Brinkman et al. [94]
7	GSE29413	H3K9me3	ESC	ChIP-seq	Karimi et al. [95]
8	E-ERAD-79	H3K4me(1,3)	ESC (WT, *Cfp1* KO)	ChIP-seq	Clouaire et al. [39]
9	GSE41440	H3K4me1, H3K27me3	MEF (WT, *Mll1* KO)	ChIP-seq	Herz et al. [33]
10	GSE44393	H3K4me3, H3K27me3	MEF (WT, *Dnmt1* KO)	ChIP-seq	Reddington et al. [59]
11	GSE39610	MBD (1A,1B,2,3,4), MECP2	ESC	ChIP-seq	Baubec et al. [16]
12	GSE34094	CTCF	ESC	ChIP-seq	Sleutels et al. [96]
13	GSE37338	Transcription	ESC	RNA-seq	Livyatan et al. [97]
14	GSE44733	Transcription	MEF (WT, *Dnmt1* KO)	RNA-seq	Reddington et al. [59]
15	GSE42836	DNA methylation	Liver, Cortex	WGBS	Hon et al. [98]

## Results

### H3K4me1, in contrast to all other active chromatin marks, is positively correlated with DNA methylation within hypomethylated regions at enhancers and promoters

The correlation between specific chromatin marks and DNA methylation has already been studied in promoters and gene coding regions [1, 20], but with insufficient focus on enhancers. Therefore, we compiled a set of 210,048 genomic sites, each of length 1 k base (kb), centered over Promoters-TSSs (+/− 500 bp of the TSS), as well as the cross-tissue putative enhancers (reported in 19 mouse cell types). We calculated the average DNA methylation of each genomic site in mouse ESCs, and split the list of genomic sites into two groups based on their DNA methylation level: hypermethylated sites (DNA methylation >50%, *N* = 186,564) and hypomethylated sites (DNA methylation ≤50%, *N* = 23,484). Hyper- and hypomethylation usually refer to increased or decreased DNA methylation without a specific boundary, and we also use these terms to simplify the presentation of our results. The 50% is not a sharp boundary and slight changes in its value do not affect our conclusions.

Within each DNA methylation group, we analyzed the correlation with DNA methylation of promoters and enhancers. While the promoters are easy to determine since they are around the TSS, the enhancers can occur in any genomic region including repeat-associated regions {Short Interspersed Nuclear Element (SINE), Long Interspersed Nuclear Element (LINE), Simple repeat, Long Terminal Repeat (LTR), DNA Transposon, Low complexity, DNA Transposon}, Intergenic, Intron, coding regions {Exon, 3’UTR, Transcription Termination Site (TTS)}, Non-coding, CpG island, and Others (merging the cases with less than 100 members, see Methods).

**Fig. 1:**
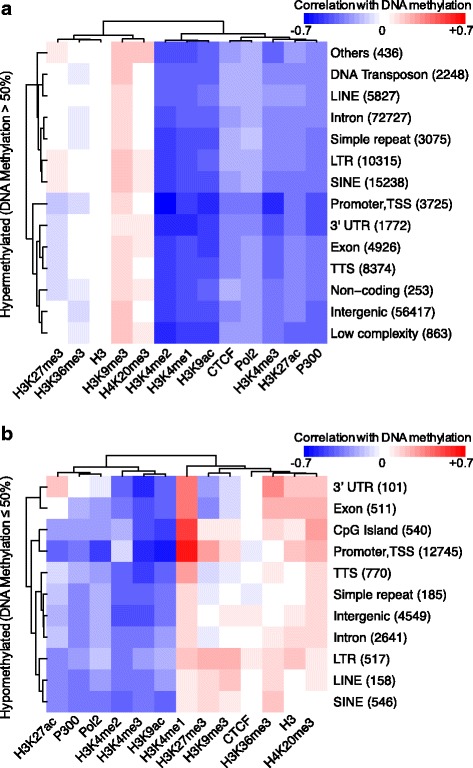
Correlation of chromatin marks and gene transcription regulators with DNA methylation in promoters and putative enhancers. The promoters are labeled as Promoter, TSS. The putative enhancers are distributed across different classes including repeat-associated regions {Short Interspersed Nuclear Element (SINE), Long Interspersed Nuclear Element (LINE), Simple repeat, Long Terminal Repeat (LTR), DNA Transposon, Low complexity, DNA Transposon}, Intergenic, Intron, coding regions {Exon, 3’UTR, Transcription Termination Site (TTS)}, Non-coding regions, CpG island, and Others. The promoters and different classes of enhancers are split into (**a**) DNA hypermethylated (DNA methylation >0.5) and (**b**) DNA hypomethylated (DNA methylation ≤0.5) groups. In each DNA methylation group, regulatory sites are classified based on their genomic location (rows). For each class, Spearman’s rank correlations, ρ, between DNA methylation of ESCs and 9 different chromatin marks, the repressive histone 3 (H3), the gene transcription marker RNA polymerase 2 (Pol2), the enhancer marker histone acetyltransferase P300, and insulator marker CCCTC-binding factor are presented in columns. Red, white and blue colors show positive, null and negative correlations, respectively

For each of the resulting 14 classes (one promoter and 13 enhancer classes), we calculated the correlation of DNA methylation with 9 chromatin marks {H3K4me1, H3K4me2, H3K4me3, H3K9ac, H3K9me3, H3K27ac, H3K20me3, H3K27me3, H3K36me3}, the repressive histone 3 (H3), the gene transcription marker RNA polymerase 2 (Pol2), the enhancer marker histone acetyltransferase P300, and the binding of the insulator marker CTCF in mouse ESCs (Fig. 1, and Table 1, rows 1, 4–7, 12).

The active chromatin marks (H3K4me1, H3K9ac, H3K4me3, H3K27ac) show negative correlations with DNA hypermethylated classes, while some repressive marks, including H3K9me3 and H3K20me3, are positively correlated (Fig. 1a). Among all the genomic regions in our study, the negative correlation with DNA hypermethylated is especially strong in Promoter-TSSs. The DNA hypomethylated sites represent a similar pattern, particularly for the active chromatin marks (H3K9ac, H3K4me3, and H3K27ac) (Fig. 1b). H3K4me1, however, exhibits an opposite pattern between DNA hyper- and hypomethylated regulatory regions: its correlation with DNA methylation is negative within all hypermethylated classes (Fig. 1a), but positive for DNA hypomethylated classes, especially within Promoter-TSSs, and within putative enhancers in CpG islands, Exons and 3’UTRs (Fig. 1b). The latter result is unexpected, since DNA methylation is generally regarded as a repressive epigenetic mark, and H3K4me1 is a hallmark of active or poised enhancers [4], hence a negative correlation is more likely between them.

According to their distance to the TSSs, the enhancers are usually classified into proximal and distal enhancers. The flanking regions of promoters are usually enriched with H3K4me1 defining proximal enhancers; however, the study of DNA methylation regulating histone methylation is more relevant in distal regions. Therefore, in order to focus our analysis on distal enhancers we excluded from the list of putative enhancers those located inside genes (promoters, exons and introns) or within a distance of less than 3 kb from the closest TSS. We found a clear anti-correlation between H3K4me1 and DNA methylation over hypermethylated distal enhancers (Additional file 1: Figure S1a), whereas the distal enhancers over genomic regions with DNA methylation lower than 50% are positively correlated between H3K4me1 and DNA methylation (Additional file 1: Figure S1b). Additionally, it is noteworthy in Additional file 1: Figure S1b the very high correlation of H3K4me1 with distal enhancers lying over CpG islands. Hence, H3K4me1 exhibits positive correlation with DNA hypermethylated enhancers in general and with DNA hypermethylated distal enhancers in particular.

Since enhancers are often shorter than 1 kb and both H3K4me1 and DNA methylation could localize to the same 1 kb element, but not necessary with local overlap, this could drive correlations between DNA methylation and H3K4me1. Therefore, we performed the above correlation analysis with window sizes of +/− 100 bp (total size 200 bp, Additional file 1: Figure S2) and +/− 200 bp (total size 400 bp, Additional file 1: Figure S3). The smaller window sizes decrease the number of promoters/enhancers, since many of them lack required number of CpGs in the smaller window to measure the DNA methylation level. Nonetheless, these results confirm the correlation between H3K4me1/H3K4me3 and DNA methylation, independently of the window size.

### H3K4me1, in contrast to all other active chromatin marks, is enriched at intermediate DNA methylation level

**Fig. 2:**
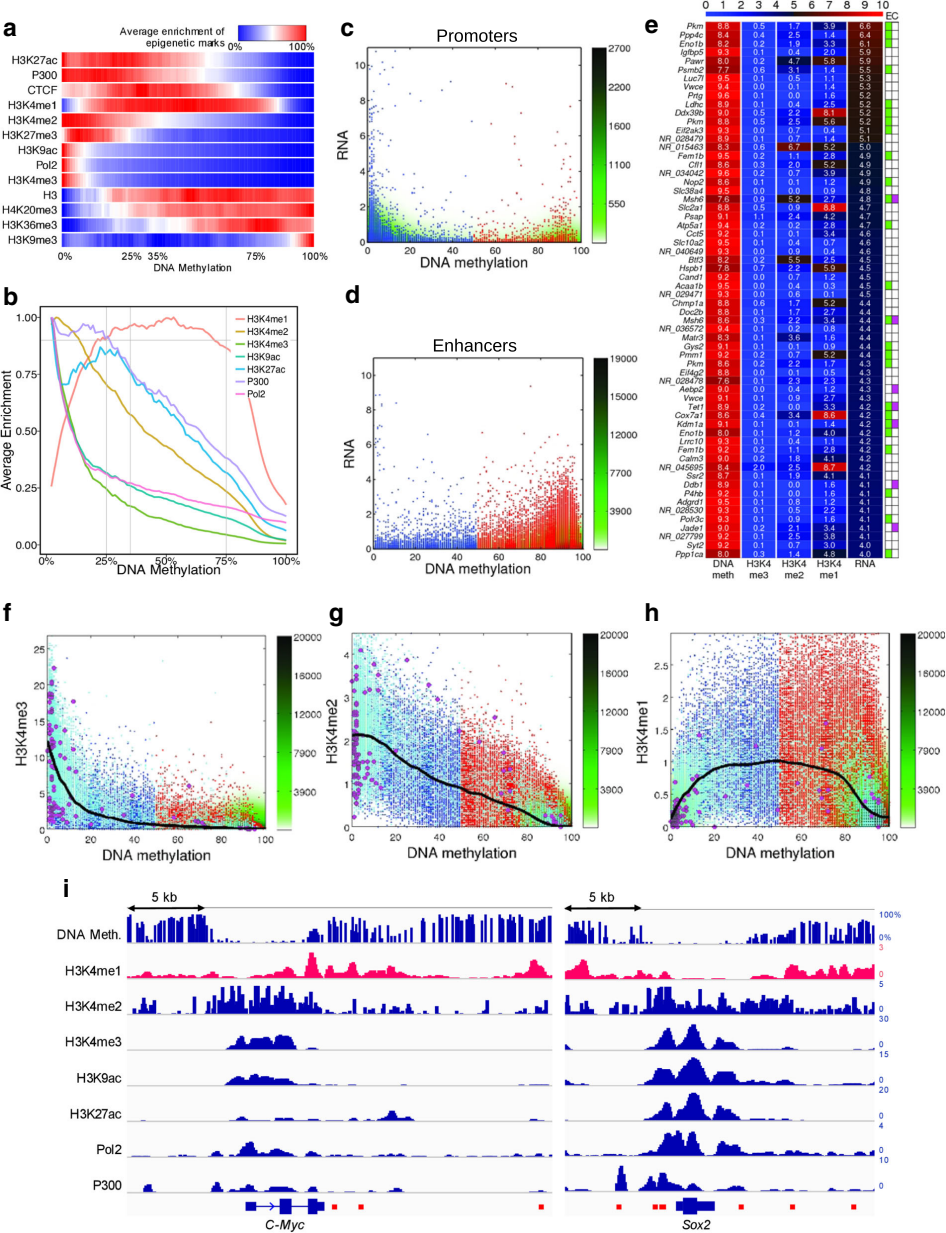
Distinct deposition of H3K4me1 from the other active chromatin marks. The regulatory sites are sorted according to their DNA methylation level in ESCs from 0 to 100% methylated. Average enrichment of different chromatin marks (rows) over sites of the same DNA methylation level are shown with (**a**) color bars and (**b**) lines (for the seven active chromatin marks). Average enrichments are scaled to have equal maximum for different marks. Pairwise scatter plots of DNA methylation versus RNA transcription for promoters (**c**) and enhancers (**d**). The scattering density is shown in green. Red and blue dots show sites with DNA methylation lower or higher that 50%, respectively. Cyan spreads show promoter sites and magenta circles show the promoters whose transcription is more than 4 in log_2_ scale. (**e**) Heat map of hypermethylated enhancers (DNA methylation >75%) and expressed transcripts (transcription >4 in log_2_ scale). To adjust the color codification, the DNA methylation, percentages are multiplied by 0.1, and H3K4me2 and H3K4me2 peaks by 5, the RNA-seq values are in log_2_ scale. Higher values correspond to redder color. The table to the right annotates Gene Ontology (GO) terms: E (Enzymatic activity) in green and C (Chromatin organization regulation) in magenta. H3K4 methylation, me3 (**f**), me2 (**g**) and me1 (**h**), enrichments within regulatory sites versus DNA methylation. Each point represents a single regulatory site. Each point represents a single regulatory site. The scattering density is shown in green. Red and blue dots show sites with DNA methylation lower or higher that 50%, respectively. Cyan spreads show promoter sites and magenta circles show the promoters whose transcription is more than 4 in log_2_ scale. The over imposed black lines mark the median of the H3K4 methylations smoothed using a robust loess regression. (**i**) DNA methylation and enrichment of the seven active chromatin marks around the *Myc* and *Sox2* gene *loci*. The location of all known putative *Myc* and *Sox2* enhancers taken from the supplemental material of Shen et al. [45] and from PHANTOM5 [46], are marked by red bars at the bottom. The y-axis represents the DNA methylation measured as the percentage of reads that support the methylated state of each CpG (estimated methylation level). For each histone mark track and for the Pol2 and P300 tracks, the y-axis represents the normalized level of ChIP-seq signal over the genomic regions

To analyze the distinct deposition of H3K4me1 over the DNA methylation landscape, we sorted the list of regulatory regions based on their DNA methylation level, and averaged the enrichments of each chromatin mark (Fig. 2a). We found that repressive chromatin marks such as H3K9me3, H4K20me3 and histone 3 (H3) are statistically significantly overrepresented in hypermethylated regions, while active chromatin marks are enriched at DNA hypomethylated promoters and enhancers (*p-*value <1e-15), i.e., the regulatory regions with DNA methylation >95% are 5-fold more enriched of H3K9me3 and simultaneously 10-fold less enriched of H3K4me3, compared to the <5% DNA methylated regions.

H3K4me1 enrichment is clearly distinct from all the other active chromatin marks (Fig. 2b). It is most enriched (0.9) at intermediate DNA methylation levels (25 - 75%), and is enrichment diminished at DNA methylation levels below 25% or above 75%, whereas H3K27ac, whose enrichment distinguishes the active from primed enhancers, is enriched in the lower range (25 - 35%) of the same intermediate DNA methylation level and decreases linearly in the higher range (35 - 75%) of the intermediate DNA methylation (Fig. 2b). Thus, when the DNA methylation of the enhancers decreases, the enhancers switch from a primed to an active state.

We studied the correlation of the signal of the three methylation states of H3K4 {me1, me2, me3} with the DNA methylation level, and found that while H3K4me2 and H3K4me3 signals anticorrelate with DNA methylation level across the whole DNA methylation range, H3K4me1 correlates positively with DNA methylation in the 0 - 50% range and negatively in the 50 - 100% range (Fig. 2f-h). We observed that DNA methylation affects RNA expression differentially promoters and enhancers. Whereas in the case of promoters, RNA expression was depleted for the middle range of DNA methylation (Fig. 2c), for the case of enhancers RNA expression was less affected for DNA methylation levels of more than 75%. We searched for non-canonically expressed enhancers, i.e., those that being highly methylated (DNA methylation >75%) are nevertheless expressed. Among them we found multiple enzymes, such as the three *loci* of the muscle pyruvate kinase (*Pkm*), lactate dehydrogenase C (*Ldhc*), glycogen synthase 2 (*Gys2*), prolyl 4-hydroxylase subunit β (*P4hb*), two *loci* of the protein phosphatase 4, catalytic subunit (*Ppp4c*), the epigenetic regulators tet methylcytosine dioxygenase 1 (*Tet1*), and jade family PHD finger 1 (*Jade1*); and transcriptional regulators such as the transcriptional repressor pro-apoptotic WT1 regulator (*Pawr*); the transcriptional and translational initiators: basic transcription factor 3 (*Btf3*) and eukaryotic translation initiation factor 4, gamma 2 (*Eif4g2*) among others (Fig. 2e).

Next, we validated our finding that in contrast to the other active chromatin marks (H3K9ac, H3K4me3, H3K4me2, H3K27ac), H3K4me1 is less enriched in both unmethylated and highly methylated regulatory regions, but overrepresented in regions with intermediate levels of DNA methylation (Fig. 2a and b), by co-localizing the DNA methylation level and histone mark signals with the known enhancer coordinates of the *Myc*/*c-Myc* and *Sox2* pluripotent genes in ESCs [45, 46] (Fig. 2i). In the case of *Myc*, the three known enhancers co-localize with peaks of high H3K4me1 signal and intermediate DNA methylation level. In the case of *Sox2*, two enhancers (5 and 6) co-localize with peaks of high H3K4me1 signal and intermediate DNA methylation level, and four enhancers (1, 2, 3 and 4) co-localize with peaks of the P300 and very low DNA methylation level.

### Neither methyl-binding proteins, nor cytosine hydroxymethylation can explain the distinct H3K4me1/3 deposition

To search for possible molecular mechanisms that explain the positive correlation between DNA methylation and H3K4me1 at hypo- to intermediate DNA methylation level at regulatory sites, we examined two conjectures: (i) Proteins with MBDs could be potential mediators of the distinct H3K4me1/3 deposition. (ii) The transition of cytosine methylation towards unmethylation through the cytosine hydroxymethylation transitory state could be associated with the H3K4me1 enrichment at intermediate DNA methylation level.

MBD proteins link to DNA through binding DNA methylated sites to some histone modifications, i.e. MBD1 forms a complex with the H3K9 methylase SETDB1, which is suggested to form stable heterochromatin histone marks over methylated DNA [47, 48]. Additionally, MBD3 is enriched at active promoters (with a positive correlation with H3K4me3) and at the enhancers of active genes that are usually H3K4me1 marked [49, 50]. Indirect interactions between MBDs and H3K4 methylation can also be hypothesized, i.e. ZIC2, an enhancer-binding factor which co-localizes H3K4me1 and the other enhancer marks (P300, H3K27ac) is shown to interact with MBD3/NURD in mouse ESCs [51]. Thus, the MBDs could be effectors of the crosstalk between DNA methylation and the H3K4me1 and H3K4me3 interaction observed here.

**Fig. 3:**
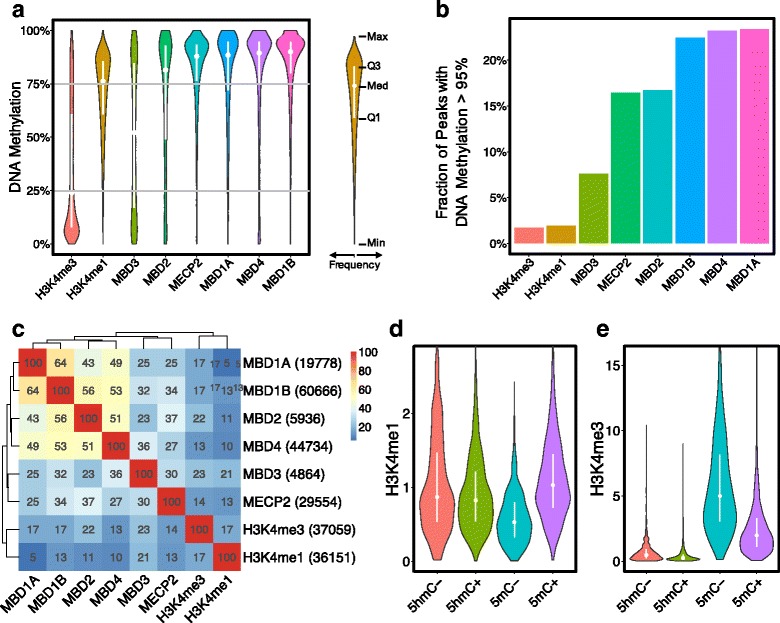
Correlation analysis enriched sites of H3K4 methylation, DNA methyl binding proteins (MBD3, MBD2, MECP2 MBD1A, MBD4 and MBD1B), DNA 5mC and 5hmC. (**a**) Violin plots of the DNA methylation distribution (y-axis) within peaks of H3K4me1, H3K4me3, and MBD proteins. The vertical white segments inside the violins connect the first (Q1) and the third quartile (Q3), and the white point represents the median (Med) of the DNA methylation level of the peaks. (**b**) Bar plot of the fraction of the highly methylated peaks (DNA methylation >95%) among all peaks of H3K4me1 and H3K4me3, and MBD binding regions. (**c**) Heat map of number of pairwise overlaps between peaks of two signals (chromatin marks or protein binding), *O*_*SiSj*_ (eq. 2), in %. The peak frequencies are shown in parentheses in the row labels. Alternations in (**d**) H3K4me1 and (**e**) H3K4me3 enrichment (y-axes) in the absence (−) or presence (+) of either 5mC or 5hmC (y-axes)

Therefore, to check the MBD effectors hypothesis we compared the chromatin immunoprecipitation sequencing (ChIP-seq) profiles of the MBD proteins for which data is available: MBD1A/B, MBD2, MBD3, MBD4 and MECP2 (Table 1, row 11) with enrichment sites of H3K4me1 and H3K4me3 in mouse ESCs (Table 1, row 4). In this analysis we included all genomic sites that showed a statistically significant peak of the chromatin marks or of the protein binding, regardless of whether such genomic sites are located within promoter/enhancer regions or not. H3K4me1 peaks occur at intermediate to high DNA methylation level, median DNA methylation (Med) = 76%, whereas the MBD proteins binding *loci* are very highly DNA methylated (Med > 90%), with the exception of MBD3 (Med = 52%) and MBD2 (Med = 81%). H3K4me3 enrichment occurs at low DNA methylation level (Med = 24%) (Fig. 3a). Such results point out lack of correlation between H3K4me3 deposition and MBD protein binding DNA methylation over all the DNA methylation ranges (low, intermediate and high), and not so obvious lack of correlation between H3K4me1 deposition and MBD protein binding DNA methylation. To resolve this case, we zoomed into the intermediate to high range of DNA methylation (50 - 100%) to check some possible correlation of MBD binding and H3K4me1 enrichment. For this purpose, we calculated the fraction of the highly methylated peaks (DNA methylation >95%) among all peaks of H3K4me1 and H3K4me3, and MBD binding regions (Fig. 3b). 10 - 20% of the MBD binding peaks populate the over 95% DNA methylation range, in contrast to only 2% H3K4me1 marks populating the same range, which rejects the possibility of overlap direct interaction between methyl-binding proteins and H3K4 methylation. We analyzed further this possibility through computing the number of all possible pairwise overlaps between peaks of two signals (chromatin marks or methyl-binding proteins) (Fig. 3c). We found that for all methyl-binding proteins there were more peak overlaps with H3K3me3 than with H3K3me1. The methyl-binding protein with highest number of overlaps with H3K4me1 is MBD3, i.e. it has a 21% of peaks overlapping with the H3K4me1 (accounting for 7592 peaks), and a 23% peaks overlapping with H3K3me3 (amounting to 8524 peaks). The other methyl-binding proteins have even less overlaps with H3K4me1 peaks (5 to 13%). These results abrogate the hypothesis of a possible connection between methyl-binding proteins and H3K4me1 deposition.

We observed that H3K4me1 is enriched at intermediate DNA methylation level, leading to the conjecture that such intermediary level might correspond to bidirectional DNA high ↔ low methylation transitions. Since it has been considered that DNA cytosine hydroxymethylation (5hmC) is an intermediate state in the process of active DNA cytosine demethylation [52], is conceivable to hypothesize that the observed intermediate DNA cytosine methylation associated with H3K4me1 enrichment might also correlate with DNA cytosine hydroxymethylation. Therefore, it is worth to study whether there is a correlation between DNA cytosine hydroxymethylation and the dynamics of the distinct H3K4me1/3 states when DNA methylation is in the transitory way to be reduced during the intermediary DNA cytosine hydroxymethylation.

To check the DNA cytosine hydroxymethylation hypothesis, we designed a method to find out which one of the DNA cytosine methylations (5mC or 5hmC), has stronger impact on the level of H3K4 methylation (H3K4me1 and H3K4me3). For this purpose, we compared alternations between a present (+) and an absent (−) state of one form of cytosine methylation (5mC or 5hmC) while the other form remains constant at a background level. Since WGBS (Whole-Genome Bisulfite Sequencing) data cannot discriminate directly between 5mC and 5hmC levels of a CpG but the sum of both DNA methylation types, we designed a method to infer 5mC from WGBS and TAB-seq (Tet-assisted bisulfite sequencing), see Eq. 1 in Methods section. We identified two groups of putative enhancers for each form of cytosine methylation (5mC or 5hmC). Each of these two groups has two subgroups, each subgroup with a similar distribution of one form of cytosine methylation working as a background but with altered level into two states (present +, or absent -) of the other form of cytosine methylation. Thus, the 5hmC alteration group consists of two subgroups {5hmC+, 5hmC-} of enhancers with significantly different 5hmC (present +, or absent -) but equal 5mC distributions, while the 5mC alteration group consists of two subgroups {5mC+, 5mC-} of enhancers with significantly different 5mC (present +, or absent -) but equal 5hmC distributions. Hence we could study the effect of the “altered” (present +, or absent -) form of DNA cytosine methylation, independently from the “background” (equal) form of methylation. We calculated the enrichment of H3K4me1 and H3K4me3 for each of the identified groups to study whether the hydroxymethylation of cytosines (5hmC) is the cause of the positive correlation between DNA methylation and H3K4me1 on DNA hypomethylated regulatory sites (Figs. 3d and e, and Table 1, rows 1, 3 and 4). We found out that alternation in 5mC levels coincides with a significant change in both H3K4me1 and H3K4me3 enrichment of regulatory sites, the H3K4me1 level increases from the group of 5mC- to the group of 5mC+ enhancers whereas the H3K4me3 level decreases from the group of 5mC- to the group of 5mC+. However, both H3K4me1 and H3K4me3 enrichments of enhancers having similar 5mC but different 5hmC are almost the same. Hence, a possible role of cytosine hydroxymethylation on H3K4me1/3 regulation is rejected and the role of cytosine methylation on H3K4me1/3 regulation is reinforced.

### DNA methylation regulates H3K4me1 - H3K4me3 seesaw

Since our previous conjectures for explaining the molecular mechanisms ruling the enrichment of H3K4me1 within DNA methylated regulatory sites were rejected, we asked the reverse question: Why H3K4me1 is not increased at DNA unmethylated regulatory sites (promoters and putative enhancers) as it could be expected for an active mark? We have already observed elevated H3K4me3 over diminished H3K4me1on DNA unmethylated regulatory sites, Particularly, the enrichment of H3K4me3 has the highest fold-change between DNA hypo- and hypermethylated regulatory sites among all active chromatin marks in this study. Hence, we hypothesized the existence of a seesaw between H3K4me1 and H3K4me3 occupancy within regulatory sites, which is controlled by DNA methylation. While both chromatin marks are depleted at DNA hypermethylated regions, the activation of this seesaw mechanism is restricted to the regulatory sites with zero to intermediate levels of DNA methylation.

**Fig. 4:**
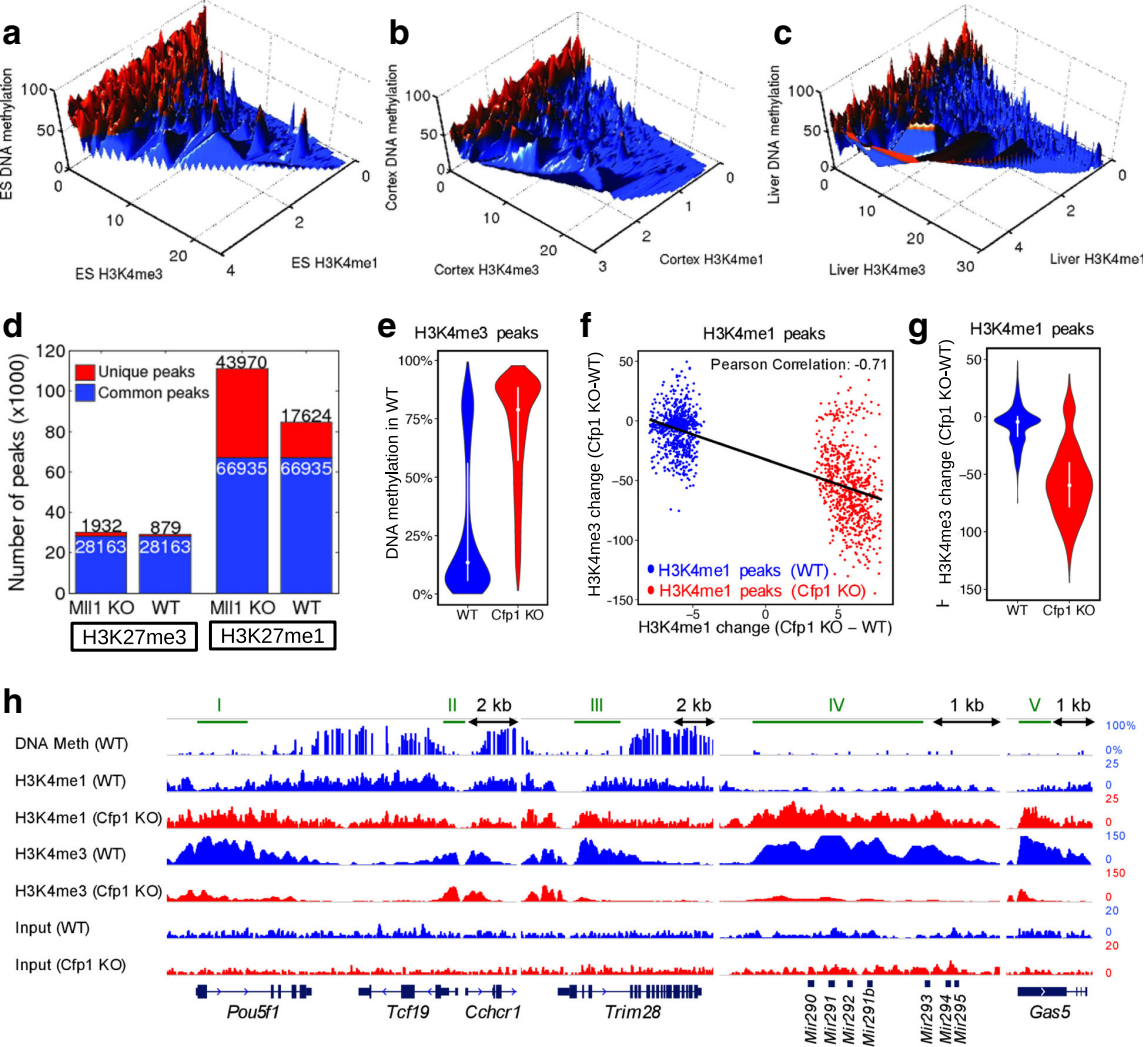
DNA methylation regulates the seesaw between H3K4me1 and H3K4me3. Surface of enrichment of H3K4me3 versus H34me1 within regulatory sites of (**a**) ESCs, (**b**) cortex, and (**c**) liver cells. Blue and red points represent regulatory sites with DNA methylation lower and higher than 50%, respectively. (**d**) Number of H3K4me1 and H3K27me3 peaks in WT and *Mll1* KO MEF cells. (**e**) Distribution of the WT DNA methylation in genomic regions specifically enriched of H3K4me3 in WT or *Cfp1* KO ESCs. (**f**) Scatter plot of the changes in H3K4me3 versus H3K4me1 enrichment (only for the H3K4me3/1 peaks with DNA methylation <50%) from WT to *Cfp1* KO cells. Blue and red points represent H3K4me1 peaks specific to WT and *Cfp1* KO cells, respectively. (**g**) Distribution of H3K4me3 changes for different H3K4me1 peaks (only for the H3K4me3/1 peaks with DNA methylation <75%) specific to WT or *Cfp1* KO cells. (**h**) DNA methylation, H3K3me1 and H3K4me3 profiles of WT (blue tracks) and *Cfp1* KO (red tracks) within several *loci*. Five genomic regions (I to V) approximately covering the gene promoters are indicated with green segments above charts. The y-axis represents the DNA methylation measured as the percentage of reads that support the methylated state of each CpG (estimated methylation level). For each histone mark track, the y-axis represents the normalized level of ChIP-seq signal over the genomic regions

We checked this hypothesis in mouse pluripotent ESCs (Fig. 4 and Table 1, rows 1 and 4). Regulatory regions with the highest H3K4me3 enrichments were DNA unmethylated and H3K4me1 decreased (Fig. 4a). In contrast, the regions with elevated H3K4me1 enrichment had higher DNA methylation but less H3K4me3. A similar analysis of cortex and liver cells (Table 1, row 15) confirmed that our finding is also true for differentiated cells (Figs. 4b and c).

### The regulation of the H3K4me1 - H3K4me3 seesaw by DNA methylation is mediated through protein CXXC DNA binding domains

The MLL1/2 and SET1A/B protein complexes responsible for deposition of H3K4me3 to the nucleosomes [42, 53, 54] share homologous CXXC subunits (CXXC7 in MLL1/2 and CXXC1 in the CFP1 component of the SET1A/B complex). These CXXC subunits are missing in the H3K4me1 depositing histone methyltransferase MLL3/4 complex [31–33]. CXXC binding domains are known to bind unmethylated CpG rich genomic regions, particularly CpG islands [39, 55]. To obtain mechanistic insights into the seesaw mechanism here proposed, we studied the influence of the presence or absence of CXXC domains on the performance of the seesaw mechanism through the computational analysis of knock out (KO) of such domains in pluripotent and differentiated mouse cells.

An expected consequence of the seesaw mechanism would be the elevation of H3K4me1 after the block of H3K4me3 in DNA hypomethylated regions. We counted the number of H3K27me3 and H3Kme1 peaks in wild type (WT) and CXXC7 (MLL1) KO from mouse embryonic fibroblast (MEF) (Table 1, row 9) and the results confirmed such prediction: the number of H3K4me1 peaks in the MLL1 KO is 31% higher (*p-*value *<*1e-15, binomial test) than in the WT cells (Fig. 4d). The frequency of H3K27me3 peaks had a minor (< 4%) difference that showed the analysis was not biased (Fig. 4d).

Next, we studied how the influence on the H3K4 methylation exerted by the CXXC1 (CFP1) component of SET1A/B in ESCs is related to DNA methylation. We used the ESC WT and *Cfp1* KO H3K4me1/3 ChIP-seq peaks from Clouaire et al. [39] (Table 1, row 8) and we co-localized them with ESC DNA methylation from Stadler et al. [56] (Table 1, row 1). We identified 8409 H3K4me3 peaks specific for WT cells and 13,184 peaks specific for *Cfp1* KO, in addition to the 78,847 common peaks between WT and *Cfp1* KO cells. The number of distal (i.e. > 5 kb distance from a TSS) H3K4me3 peaks is significantly increased in *Cfp1* KO cells (11,352 peaks in *Cfp1* KO, and 4663 in WT).

To study how DNA methylation influences the lack of unmethylated CpG CXXC binding domains (*Cfp1* KO) on H3K4me3, we selected the peaks with at least 2% CpG content. We found a significant change between the DNA methylation of the WT and *Cfp1* KO specific H3K4me3 peaks, median DNA methylation of 13 and 79%, respectively (Fig. 4e). Since WT peaks are restricted to DNA hypomethylated regions, this finding suggests that the ablation of *Cfp1* allows the appearance of H3K4me3 peaks in DNA hypermethylated regions. This suggestion is in agreement with previous studies [39, 55]. We do not exclude, however, the possibility of reduced activity of DNA methyltransferases and global hypomethylation in Cfp1-KO cells as reported previously [57].

Additionally, we identified 7638 H3K4me1 peaks specific to WT, 8234 specific to *Cfp1* KO cells, and 116,373 H3K4me1 peaks in both cell types. Since we hypothesized that there is a seesaw mechanism between H3K4me1 and H3K4me3 within low to intermediate DNA methylation, we focused our analysis on peaks with DNA methylation below 50%. The WT-specific H3K4me1 peaks have significantly higher H3K4me1, but lower H3K4me3 enrichment than the *Cfp1* KO specific peaks, and vice versa (Fig. 4f, g, *p*-value <1e-15). Particularly, H3K4me1 enrichment shows a significantly negative correlation (Pearson’s correlation coefficient *r =* −0.71) with H3K4me3 enrichment, i.e. within low to intermediate DNA methylation increased H3K4me1 levels encompassed with reduced H3K4me3 levels when *Cfp1* is knocked out which further confirms the seesaw model (Fig. 4f), thus reduced H3K4me3 (due to Cfp1KO) elevates the seesaw towards H3K4me1.

To illustrate how the co-localization of the H3K4me1 and H3K4me3 signals is influenced by the disruption of CFP1, we studied the genomic region around the master of pluripotency transcription factor *Pou5f1/Oct*4 (Fig. 4h). The unmethylated promoter of *Pou5f1* (region I) is depleted of H3K4me1 and enriched of H3K4me3 in WT cells, while the *Cfp1* KO cells are enriched of H3K4me1 and depleted of H3K4me3 in the same *loci*. Similarly, the transcriptional intermediary factor *Trim28*, the pluripotency-associated *Mir290* cluster of microRNAs, and the non-coding RNA gene *Gas5* (regions III-IV) show elevated H3K4me1 coinciding with depleted H3K4me3 after *Cfp1* KO. These results show how the disruption of CFP1, alters the balance between H3K4me1 and H3K4me3. The promoter shared between *Tcf19* (Transcription factor 19) and *Cchcr1* (Coiled-coil α-helical rod protein 1) transcribed in opposite directions (region II), however, it shows almost similar chromatin patterns in WT and *Cfp1* KO cells.

### DNA hypomethylation causes H3K4me3 enrichment and aberrant gene expression

**Fig. 5:**
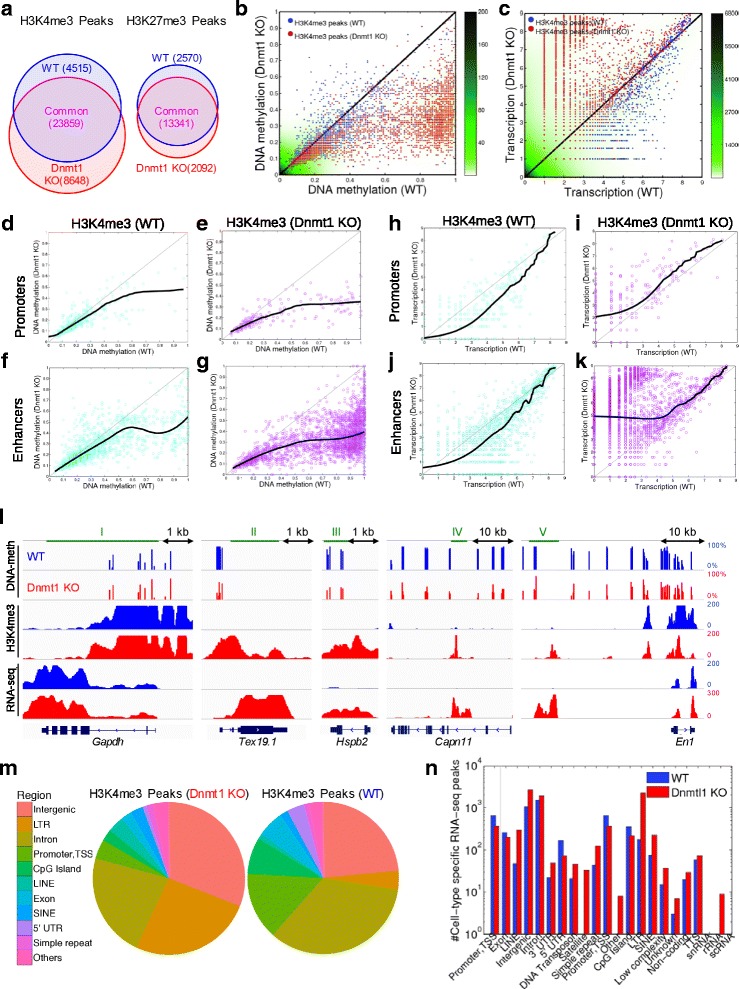
DNA hypomethylation is followed by H3K4me3 enrichment and activates transcription. (**a**) Venn diagrams of number of H3K4me3 (left) and H3K27me3 (right) peaks in WT and *Dnmt1* KO MEF cells, in blue and red, respectively. (**b**) Scatter plot of DNA methylation profiles in *Dnmt1* KO versus WT cells. (**c**) Scatter plot of RNA-Seq transcription profiles of *Dnmt1* KO versus WT. The scattering density is shown in green. (**d**) Scatter plot of DNA methylation profiles in *Dnmt1* KO versus WT cells for H3K4me peaks over promoters of WT cells. (**e**) Scatter plot of DNA methylation profiles in *Dnmt1* KO versus WT cells for H3K4me peaks over promoters of Dnmt1 KO cells. (**f**) Scatter plot of DNA methylation profiles in *Dnmt1* KO versus WT cells for H3K4me peaks over enhancers of WT cells. (**g**) Scatter plot of DNA methylation profiles in *Dnmt1* KO versus WT cells for H3K4me peaks over enhancers of Dnmt1 KO cells. (**h**) Scatter plot of transcriptomics profiles in *Dnmt1* KO versus WT cells for H3K4me peaks over promoters of WT cells. (**i**) Scatter plot of transcriptomics profiles in *Dnmt1* KO versus WT cells for H3K4me peaks over promoters of Dnmt1 KO cells. (**j**) Scatter plot of transcriptomics profiles in *Dnmt1* KO versus WT cells for H3K4me peaks over enhancers of WT cells. (**k**) Scatter plot of transcriptomics profiles in *Dnmt1* KO versus WT cells for H3K4me peaks over enhancers of Dnmt1 KO cells. (**l**) DNA methylation, H3K4me3 and transcription in several *loci* of WT (blue tracks) and *Dnmt1* KO (red tracks) MEF cells. Green bars above the gene map locate the CpG islands. The y-axis represents the DNA methylation measured as the percentage of reads that support the methylated state of each CpG (estimated methylation level). For each histone mark track, the y-axis represents the normalized level of ChIP-seq signal over the genomic regions. (**m** ) Pie charts of the genomic structural composition of the H3K4me3 peaks *loci* specific to WT and *Dnmt1* KO cells. (**n**) Number of specific RNA-seq peaks in WT and *Dnmt1* KO cells

We have provided several lines of evidence showing that the seesaw mechanism between H3K4me1 and H3K4me3 is regulated by DNA methylation. However, the biological impact of such regulation still needs to be identified. It is also important to determine whether this regulatory function of DNA methylation is a specific property of pluripotent cells or whether it exists also in differentiated cells. Therefore, we studied MEF cells in absence (KO) or presence (WT) of *Dnmt1,* the key maintainer of DNA methylation after cell division (Fig. 5 and Table 1, rows 2, 10 and 14). In addition to 23,859 common H3K4me3 peaks in *Dnmt1* WT and KO cell types, we found a gain of 8648 (30%) of genomic *loci* which were H3K4me3-enriched specifically in the *Dnmt1-*KO cells. This is almost twice the number of specific peaks of WT cells (4515 WT-specific) (Fig. 5a). Furthermore, the number of H3K27me3 peaks had a modest change (3%) between cell types, which confirms that the results were not cell type dependent. Similar to *Cfp1* KO cells, there were significantly more frequent distal H3K4me3 peaks specific for *Dnmt1* KO cells (*N* = 5652) compared to the WT specific (*N* = 1516) (Additional file 1: Fig. S4).

The DNA methylation pattern is significantly different between the specific peaks for each cell type, WT and *Dnmt1* KO (Fig. 5b, Additional file 1: Fig. S1). The WT-specific H3K4me3 peak locations are hypomethylated in both WT and *Dnmt1* KO cells (21 and 18% median DNA methylation, respectively). In contrast, *Dnmt1* KO-specific H3K4me3 peaks show significant loss of DNA methylation after *Dnmt1* KO (28%), while they are hypermethylated in WT (77% median DNA methylation).

Presence of H3K4me3 peaks coincides with a major shift in transcription (Fig. 5c). Among *Dnmt1* KO-specific H3K4me3 peaks, up-regulated transcribed regions in the same cell type (compared to the WT cells) are 21 times more frequent than down-regulated ones (*N* = 2931 and 139 respectively, minimum 2-fold change in transcription). Similarly, WT- specific H3K4me3 peaks with more than 2-fold up-regulation in the same cells (compared to *Dnmt1* KO) are 7.5 times more frequent than down-regulated regions (*N* = 1064 and 141, respectively).

We analyzed how the H3K4me3 peaks for each cell type, WT and *Dnmt1* KO, split between enhancers and promoters. At DNA methylomics level, the WT-specific H3K4me3 peaks are under-methylated in *Dnmt1* KO samples in relation to WT samples both in promoters (Fig. 5d) and enhancers (Fig. 5f). Interestingly, in the case of enhancers, there is a depression of DNA methylation in the *Dnmt1* KO samples for the DNA methylation level around 75% of the WT samples (Fig. 5f). The *Dnmt1* KO-specific peaks are slightly more under-methylated over promoters (Fig. 5e) than over enhancers (Fig. 5g), with a high dispersion of DNA methylation in *Dnmt1* KO samples in the same *loci* of enhancers in which the WT samples are highly DNA methylated (Fig. 5g). At transcriptomics level, the expression behavior in WT-specific H3K4me3 peaks over promoters (Fig. 5h) and over enhancers (Fig. 5j) is very similar. In both cases the transcription in WT samples is up-regulated in relation to the transcription in *Dnmt1* KO samples. Interestingly, in the case of *Dnmt1* KO-specific H3K4me3 peaks there is a strong dichotomy in the transcription behavior of enhancers (Fig. 5i) and promoters (Fig. 5k). In both cases, the expression is very similar in *Dnmt1* KO and WT samples for expression level higher than 4 (in log_2_ scale). However, for low transcription levels, the transcription is up-regulated in *Dnmt1* KO samples in relation to WT samples in enhancers (Fig. 5k).

We studied how this observation at genomics level translates into the co-localization of signals at *loci* of four cell-type specific genes (*Tex19.1*, *Hspb2*, *Capn11*, *En1*) and one house housekeeping gene, *Gapdh* (Fig. 5i). Both epigenetic (DNA methylation and H3K4me3) and transcriptional patterns of *Gapdh* (region I) are similar in WT and *Dnmt1* KO cells. In contrast, the pluripotency-associated gene *Tex19.1* that is specifically active in ES, placenta and germ cells [58] loses DNA methylation (from 100% to 25–75%) in its promoter in *Dnmt1* KO cells. This is supported by the fact that the number of CpGs with 75–100% methylation is reduced to almost zero at genomics scale in *Dnmt1* KO cells [59]. The DNA methylation loss is coincident with H3K4me3 enrichment and downstream ectopic expression in MEF cells (region II). Same scenario develops at the Heat Shock Protein Family B (Small) Member 2 coding *Hspb2* gene, normally expressed in muscle and heart (region III). Region IV is an intronic long terminal repeat (LTR) located within the spermatogenic-specific Calpain 11 coding gene *Capn11*. It is silent in WT MEFs, H3K4me3 enriched and transcribed after being hypomethylated in *Dnmt1* KO cells, although the *Capn11* gene itself is silent in both cell types. An intergenic region upstream of the neural specific Engrailed Homeobox *En1* coding gene is also shown to undergo DNA hypomethylation, H3K4me3 enrichment and active transcription in *Dnmt1* KO cells (region V).

We compared the genomic location of H3K4me3 peaks specific to WT and *Dnmt1* KO cells (Fig. 5m). The *Dnmt1* KO-specific H3K4me3 peaks were overrepresented within retroelements including LTR, LINE (long intergenic non-coding elements) and SINE (short intergenic non-coding elements). Exons, promoters and distal CpG-rich regions were elevated for WT peaks. This finding was confirmed by profiling RNA-seq peaks specific to WT and *Dnmt1* KO cells. LTR, LINE and SINE elements were significantly overrepresented in KO cells, while WT cells showed transcription enrichment within introns, intergenic regions and LTRs (Fig. 5n).

## Discussion

We analyzed the crosstalk between DNA methylation and different chromatin marks over a broad range of regulatory regions including putative enhancers and promoters. Intriguingly, in contrast to the expected significantly negative correlation between DNA methylation and active chromatin marks, we found that H3K4me1 enrichment has significantly positive correlation with intermediate (in the range between 25 and 75%) DNA methylation at regulatory regions. Existing reports about H3K4me1 and DNA methylation claim both positive [21, 60] and negative [15–17] correlations. Our results re-conciliate the two seemingly contradictory observations zooming into the less studied fuzzy intermediate range between the wide-used extreme hyper and hypo DNA methylation states.

We observed anti-correlation between H3K4me1 and H3K4me3 enrichment at low (0 - 25%) and intermediate (25 - 75%) DNA methylation. While a negative correlation between an active epigenetic mark (H3K4me1) and a repressive one (DNA methylation) at high DNA methylation (>75%) seems acceptable, we tried to uncover the mechanism responsible for the anti-correlation between H3K4me1 and H3K4me3 within low and intermediate (0 - 75%) DNA methylated regulatory regions. We hypothesized “seesaw” dynamics between H3K4me1 and H3K4me3 in the 0 - 75% DNA methylation range: while the enrichment of one mark rises up, the enrichment of the other drops down. DNA methylation discriminates between enhancers and promoters, marked by H3K4me1 and H3K4me3, respectively: low methylated regions are H3K4me3 enriched, while those with intermediate DNA methylation levels are progressively H3K4me1 enriched. Additionally, the enrichment of H3K27ac, distinguishing active from primed enhancers, follows a plateau in the lower range of the intermediate DNA methylation level (25 - 35% DNA methylation), corresponding to active enhancers, and decreases linearly in the higher range of the intermediate DNA methylation (35 - 75%). Thus, the decrease of the DNA methylation switches smoothly the state of the enhancers from a primed to an active state.

Although simultaneous mono- and trimethylation of a single H3K4 are mutually excluded, different cells of a population can have different chromatin marks at the same genomic region, and such marks are dynamically changed through the enzymatic activity of methylases and demethylases. Therefore, we use the term “seesaw” rather than “mutual exclusion” to define such mechanism, which includes a balanced state with both marks enriched at lower levels.

The H3K4me1-H3K4me3 seesaw mechanism controlled by DNA methylation is valid for both pluripotent and differentiated cells, i.e. it is not cell type-specific. We scrutinized whether DNA methylation has a mechanistic function in the discrimination of H3K4me1 from H3K4me3 marked regulatory sites. While low and intermediate DNA methylated regions of WT ESCs are depleted of H3K4me3 peaks, knocking out of the H3K4me3 methyltransferase *CxxC1* domain of *Cfp1* increases the H3K4me3 enrichment in these regions. This can be linked to reduced DNA methylation of these regions after decreased DNA methyltransferase level in *Cfp1* KO cells [61]. Additionally, unmethylated CpG-rich regions are shown to be sufficient for CFP1 binding and H3K4me3 enrichment [55], hence SET1A/B complex can be deficient of unmethylated CpG recognition sites in absence of *Cfp1* that can result in H3K4me3 enrichment of even hypermethylated regions. Both possibilities suggest an active function of DNA methylation in regulating H3K4me3 deposition.

Reports implicitly confirm that blocking H3K4me3 would result in enriched H3K4me1. The WD (glycine-histidine) repeat domain 5, *Wdr5*, a core member of mammalian H3K4me3 methyltransferases, interacts with H3K4me2 and mediates transition to H3K4me3 [62]. Immunoblot of *Wdr5* knockdown ESCs shows enriched H3K4me1 in response to depleted H3K4me3, which is due to increased DNA demethylation of H3K4me2 [63]. Furthermore, H3K4me3 enrichment coincides with H3K4me1 depletion after knock down of the histone demethylase *Kdm5c* [64]*.* The same report demonstrates that H3K4me1 is depleted at H3K4me3 peak summits. H3K4me1 peaks have higher frequency of in absence of the H3K4me3 methyltransferase domain CXXC7 of Mll1 [33]. Analysis of MEF cells in absence of *Dnmt1* provides further evidence of the functional role of DNA methylation in differentiated cells. Significant loss of DNA methylation within H3K4me3 peaks of *Dnmt1* KO cells compared to the WT MEF shows that DNA hypomethylation is a precondition for H3K4me3 deposition.

Switching between H3K4me1 and H3K4me3 discloses the role of DNA methylation in discriminating promoters and enhancers. H3K4me3 is shown to facilitate access and assembly of the RNA polymerase 2, Pol2, as well as to promote transcriptional initiation through binding of TFIID [64]. On one hand, transcriptional activity is also shown to influence H3K4me3 enrichment [65]. Our results indicate a dramatic transcriptional activity coincident with H3K4me3 enrichment in consequence of DNA hypomethylation in *Dnmt1* KO cells, which is not limited to gene coding regions but also overrepresented within non-coding and intergenic regions, particularly the retroelements. On the other hand, H3K4me1 is specifically recognized by a number of chromatin-interacting proteins [66] and is also shown to guide a pioneer transcription factor *Foxa1* for initiating enhancer complex formation. Depleted H3K4me1 by overexpression of H3K4 demethylase is followed by abrogated recruitment of the transcription factor [67], suggesting a causal role for H3K4me1 in enhancer priming.

**Fig. 6:**
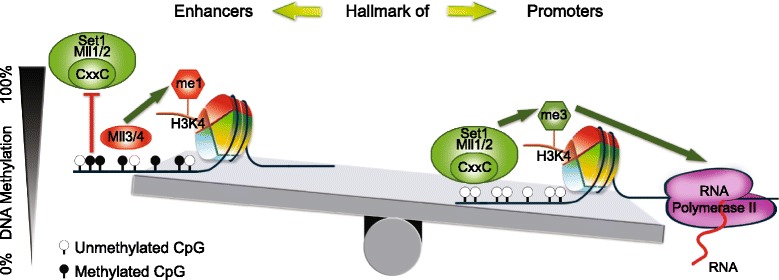
Scheme of how DNA methylation drives the seesaw mechanism between H3K4me1 and H3K4me3. CXXC binding domains including CFP1 and MLL1/2 are bound to unmethylated CpGs (right) and lead deposition of H3K4me3 (promoter mark), which results in RNA transcription. Increased DNA methylation (left) prevents binding of these CXXC domains, and the free nucleosomes can be bound by MLL3/4, which are not sensitive to methylation level, and transfer chromatin the enhancer mark H3K4me1. Decreased level of H3K4me3 due to DNA methylation coincides with a seesaw elevation of H3K4me1 and this is the mechanism behind positive correlation between DNA methylation and H3K4me1

We propose that the seesaw mechanism operates as follows: DNA unmethylated CpG-rich regions provide the basis for H3K4me3 methyltransferases (i.e. SET1A/B, MLL1/2) to bind and functionally mark the area as a promoter. This can be done by increasing the conversion of H3K4me1/2 to H3K4me3 that results in increased H3K4me3 in parallel with decreased H3K4me1, which leads to the seesaw mechanism (Fig. 6). The intermediate DNA methylation levels can reduce the binding of the CpG sensitive CXXC domain of H3K4me3 methylases, while giving access to CXXC-free H3K4me1 methylases, which results in limited conversion of H3K4me1 to H3K4me3, and marks the *locus* as enhancer by H3K4me1 enrichment. This mechanism driven by H3K4 methyltransferases is complementary to the regulatory role of H3K4 demethylases Kdm5b/c in the discrimination between promoters and enhancers [64, 68]. Thus the H3K4me3 methyltransferases and the H3K4 demethylases make possible the reversible seesaw between enhancers and promoters.

The activation of the seesaw mechanism occurs for low to medium DNA methylation levels. When DNA methylation is high, both H3K4me1 and H3K4me3 are low, marking an inactive genomic region. When DNA methylation decreases to intermediate level, H3K4me1 is high and H3K4me3 is low, marking an enhancer region. Finally, when DNA methylation decreases to a low level, H3K4me1 is low and H3K4me3 is high, marking a promoter region. We can summarize these observations into a rule of thumb of one-out-of-three methylation marks: “In each genomic region only one out of the following three methylation marks {DNA methylation, H3K4me1, H3K4me3} is high: if it is DNA methylation, the region is inactive, if it is H3K4me1, the region is an enhancer, and if it is H3K4me3, the region is a promoter”.

## Conclusions

To explain H3K4me1 depletion at high levels of DNA methylation, we suggest two possible mechanisms: (I) A passive mechanism, in which the heterochromatin structure of the genome that is incorporated with stable hypermethylation [69] can make chromatin inaccessible for many DNA or chromatin-bound proteins, TFs and potentially the H3K4me1 histone methyltransferases. (II) An active mechanism in which the recruitment of TFs by H3K4me1 leads to DNA hypomethylation and enhancer priming [67]. Interestingly, binding of some TFs causes DNA hypomethylation at low to intermediate levels in the population [1, 56], which is in agreement with our observation of enriched H3K4me1 at intermediate DNA methylation.

Additionally, our findings suggest a potential mechanism for inheritance of histone codes, particularly H3K4 methylation, during cell division: While the machinery maintaining DNA methylation during cell division is well-studied [70], little is known about how histone codes are inherited by the daughter cells. H3K4me3 methylase is shown to remain associated with the newly replicated DNA through unknown mechanisms during cell division, although the histones carrying the H3K4me3 mark are replaced by unmethylated histone 3 (H3) after DNA replication [71]. We suggest that re-established DNA methylation of the nascent DNA could provide complementary information on how H3K4 methylases transmit the H3K4 methylation patterns from the parent to the daughter cells in a more reliable manner. This would help to establish a framework for the inheritance of chromatin marks and a genomic map of promoters and enhancers that are inherited by DNA methylation.

The DNA methylation regulating a H3K4me1 - H3K4me3 seesaw mechanism has implications in developmental biology, cellular reprogramming, cancer and aging. It changes the balance in differentiation- and pluripotency-related genes. The promiscuous DNA hypomethylation of cancer cells can disrupt the normal deposition pattern of promoter and enhancer chromatin marks, followed by aberrant transcription of silent genes and non-coding regions. The disturbance in DNA methylation can change also the balance between enhancers and promoters in aging related genes.

## Methods

### Data sources

ChIP-seq data of genome-wide maps of chromatin marks, Pol2 and gene expression regulators, RNA-seq and different forms of bisulfite sequencing (Bis-seq) including Whole-Genome Bisulfite Sequencing (WGBS), Reduced Representation Bisulfite Sequencing (RRBS) and Tet-assisted bisulfite sequencing (TAB-seq), used for measurement of DNA hydroxymethylation 5hmC) were obtained from several GEO or ArrayExpress datasets (Table 1). The coordinates of putative enhancers of 19 mouse tissues and cell types were taken from Tan et al. [72].

### NGS data preprocessing

The mouse reference genome assembly mm9 was used for the whole analysis, and the University of California, Santa Cruz (UCSC) liftOver tool was applied for address conversion of some datasets already aligned to mm8. We developed a pipelined script to download next-generation sequencing data in SRA or other available formats, converted them to fastq format, aligned them using Bowtie2 [73] and identified statistically significant peaks compared to the whole cell extract (WCE) inputs when available. The processing of the data has been performed as follows: The raw fastq files were aligned to the reference genome using Bowtie2, and then converted to the genome coverage wiggle format using a pipeline of several commands including bamToBed, genomeCoverageBed, bedGraphToBigWig and finally bigWigToWig commands of BEDTools [74] and UCSC Genome Browser toolkits [75]. We then used MACS2 [76] for peak finding and MAnorm [77] for normalization of genome coverage data. Hence the genome coverage values that are depicted in the figures are the normalized total number of NGS reads that are aligned to each genomic region.

### DNA methylation data processing

To assess the degree of DNA methylation of each CpG, we used our parallel processing pipeline software for automatic analysis of bisulfite sequencing data (P3BSseq) [78]. We define a CpG as 100% methylated when all the reads that are aligned to this CpG in a genomic region are 100% methylated (the reads in the CpG *loci* are of the form CpG rather than TpG that is a result of C → T conversion for unmethylated CpGs following Sodium-bisulfite treatment). We set a minimum read CpG coverage criterion, keeping only the reads having at least 10 CpG dinucleotides with coverage of minimum 5× in the bisulfite sequencing (Bis-seq) data. The CpG dinucleotides with minimum 5-reads coverage were kept in each Bis-seq data, and the average methylation ratios over CpGs inside the 1 kb window were assigned as DNA methylation level of each site.

WGBS data cannot discriminate directly between 5mC and 5hmC levels of a CpG but the sum 5×mC_WGBS_ of 5mC and 5hmC, i.e. 5×mC_WGBS_ = 5mC + 5hmC. However, since TAB-seq measures specifically the 5hmC level of each CpG, we estimate the 5mC level of a CpG by subtracting the TAB-seq measured 5hmC_TAB_ ratios [45, 72, 79–81] from the total DNA methylation level (5mC + 5hmC measured in WGBS experiments) as

1 $$ 5\mathrm{mC}=5{\mathrm{xmC}}_{\mathrm{WGBS}}-5{\mathrm{hmC}}_{\mathrm{TAB}} $$

which allows us to evaluate specifically 5mC ratios at single-base resolution. To discriminate CpGs with 5hmC from those that do not have 5hmC (those that have only 5mC), we performed (Eq. 1) calculation only on CpGs with significantly reliable 5hmC levels (False Discovery Rate (FDR) < 0.05 and Phred quality score ≥ 20).

### Assessment of the enrichment of epigenetic marks in promoters and enhancers

When studying the difference of enrichment of the several epigenetic marks between promoters and enhancers, promoter regions are relatively easy to define based on the position of the transcription start sites (TSSs). However, enhancers do not have well defined positions, and they can occur in almost any genomic region. Therefore, in order to obtain a comprehensive picture of the discrimination of epigenetic marks between promoters and enhancers, in our results we re-annotated the enhancer positions into 20 different genomic categories. Thus, we created a draft list of 428,297 non-overlapped 1 kb genomic sites centered over TSSs (−/+ 500 bp of the TSS) of genes of the National Center for Biotechnology Information (NCBI) Reference Sequence Database (RefSeq), and cross-tissue putative enhancers of 19 mouse cell types [45, 72, 79–81].

Since the enhancers can occur in any genomic region, we furthermore mapped them into 20 different categories: including repeat-associated regions {Short Interspersed Nuclear Element (SINE), Long Interspersed Nuclear Element (LINE), Simple repeat, Long Terminal Repeat (LTR), DNA Transposon, Low complexity, DNA Transposon, Satellite}, Intergenic, Intron, non-coding, CpG island, and coding regions {Exon, 5’UTR, 3’UTR, and transcription termination site (TTS)}, regions associated with different types RNA species {rRNA, scRNA, snRNA, tRNA}, and regions with “Unknown” annotation. The TSSs are 1 kb genomic sites centered (−/+ 500 bp) over the TTS. Additionally, we have created the category “Others”, that appears across the different results. In this category, we merged the cases with less than 100 members. From the initial non-overlapped 1 kb genomic sites, we filtered 210,048 sites having at least 10 CpG dinucleotides with minimum 5x coverage in the Bis-seq data. We established a feature *s* × *r*matrix *M* with the *s =* 210,048 sites in the rows, and different *r =* 13 gene regulation features including epigenetic marks {H3K27me3, H3K36me3, H3K9me3, H3K20me3, H3K4me2, H3K4me1, H3K9ac, H3K4me3, H3K27ac}, protein bindings {P300} and other genomic features {H3, CTCF, Pol2} in the columns. Enrichments of chromatin marks within each site were calculated as the average depth of reads within the 1 kb window.

### Correlation analysis of epigenetic marks with DNA methylation

All regulatory sites used in this study were classified based on the genomic structure using the annotatePeaks.pl script of the HOMER suite [73, 82]. For each of the 21 classes (one promoter and 20 enhancers classes) we computed the Spearman’s rank correlation coefficients ρ between the DNA methylation and the enrichment of 13 gene regulation features {H3K27me3, H3K36me3, H3K9me3, H3K20me3, H3K4me2, H3K4me1, H3K9ac, H3K4me3, H3K27ac, P300, H3, CTCF, Pol2}, creating a correlation *c* × *r* matrix *R* with the *c =* 21 classes (one corresponding to promoters and 20 to enhancers) in the rows, and different *r =* 13 gene regulation features in the columns. Next, all sites were sorted into 100 bins according to DNA methylation levels (i.e., bin_1_included those sites with DNA methylation level between 0 and 1%) and split into two matrices of correlations, one, *R* ^*Hyper*^ with DNA hypermethylated sites (DNA methylation >50%), and another, *R* ^*Hypo*^ with DNA hypomethylated sites (DNA methylation ≤50%). The results were represented in heat maps after hierarchical clustering of rows and columns of the matrices of correlation *R* ^*Hyper*^ and *R* ^*Hypo*^. For each of the 13 gene regulation features, the enrichments were averaged on all sites assorted in the same bin, and the results were linearly scaled between 0 and 1. The Integrative Genomics Viewer (IGV) was used for *locus*-specific representation of ChIP-seq and DNA methylation data [83].

### Peak analysis of methyl-binding proteins and chromatin marks

We used MACS [84] to calculate the fraction of peaks with DNA methylation level above 95% over the total number of peaks. Additionally, peaks of each pair of signals were compared to find overlaps. Two peaks *p*_S*i*_ and *p*_S*j*_ of two different signals S*i* and S*j*, were considered overlapped if some genomic region (even as small as a single nucleotide) was included in both. Thus we define an overlap binary variable *o*_*SiSj*_, equal to 1, if *p*_S*i* ∩_ *p*_S*j*_ ≥ 1, and 0, otherwise. For each pair of signals S_*i*_, S_*j*_, with #*p*_S*i*_ and #*p*_S*j*_ number of peaks, respectively, we calculated their percentage of overlap *O*_*SiSj*_ as the number of overlapped peaks #*o*_*SiSj*_ divided by the number of peaks of the signal with smaller number of peaks, in %, i.e.

2 $$ {O}_{SiSj}=100\#{o}_{SiSj}/\min\ \left(\#{p}_{\mathrm{S}i},\#{p}_{\mathrm{S}j}\right) $$

and represented it in a hierarchical clustered heatmap.

### Discrimination between the impact of DNA 5mC and 5hmC on H3K4 methylation

To study which of the DNA cytosine methylations (5mC or 5hmC) have stronger impact on the level of H3K4 methylation, we modeled such impact with probability theory. We observed initially that the 5hmC level (measured by TAB-seq) is gained on putative enhancers that have also higher 5mC levels (estimated by Eq. 1), hindering to consider 5mC or 5hmC as independent variables. Assuming *H3K4me1* and *H3K4me3* to be the probabilistic events of significant alternations in H3K4me1 and H3K4me3, respectively, and *5mC* and *5hmC* as the events of change in 5mC and 5hmC levels, respectively, we compared the conditional probabilities *P*(*H3K4me1|5mC*), *P*(*H3K4me3|5mC*), *P*(*H3K4me1|5hmC*), and *P*(*H3K4me3|5hmC*). Therefore, we computed the conditional probability of either H3K4me1 or H3K4me3 as a response of the 5hmC as the variable, under fixed 5mC distribution, and vice versa, 5mC as the variable, under fixed 5hmC distribution. Namely, to discriminate the possible relationship between the H3K4me1 and H3K4me3 chromatin marks and 5mC versus 5hmC, we compared alternations of one form of cytosine methylation (5mC or 5hmC) when the other form was constant (5hmC or 5mC). This is a challenging task since alternations in 5hmC is usually coincident with changes in 5mC level. To address this issue, we used a probabilistic approach to identify two groups of putative enhancers to compare for each form of cytosine methylation, (4 groups in total). Each pair of enhancer groups had a similar distribution of one form of methylation (called “background”), but altered level of the other form of methylation (“altered”). Hence, we could study the possible effect of the “altered” form of methylation independently from the “background” form of methylation.

To study the interplay between H3K4me1 or H3K4me3, and 5hmC as the altered methylation (with 5mC as the background), we built the 5hmC altered group considering two groups of enhancers {5hmC+, 5hmC-} with significantly different 5hmC but equal 5mC distributions. The first group, representing the presence of the 5hmC signal called 5hmC+, consists of 2501 putative enhancers with a minimum of 20 CpG dinucleotides and an average of 5hmC between 15 and 30% within a 1 kb window. The second group, representing the absence of 5hmC signal is called 5hmC-, has the same number of putative enhancers as the 5hmC+ group, and the same minimum of 20 CpGs but with an average of 5hmC 0% within a 1 kb window. To eliminate the possible effect in the 5mC background of 5mC alternations between the two groups, for each enhancer in the 5hmC+ group we selected an enhancer with the constraints of the other group (20 CpGs and 0% 5hmC) in such a way that the 5mC levels of the two enhancers were the closest possible, thus ensuring similar 5mC background distribution in the two groups. Thus, both groups have mean 5mC equal to 68% (*p-*value = 1). Still, 5hmC levels were significantly different. The mean 5hmC level in 5hmC+ and 5hmC-group was 17 and 0%, respectively (*p-*value <1e-15). We did not select the 5hmC+ group from higher levels of 5hmC due to the lack of sufficient number of enhancers fulfilling the criteria for comparison of the two group.

A similar approach was used to study the interplay of either H3K4me1 or H3K4me3 with 5mC, under fixed 5hmC. The 5mC-presence group, 5mC+, consisted of putative enhancers with 5mC between 15 and 30% (the same range used for the 5hmC+ group), while for the 5mC-absence group, 5mC-, we used a slightly relaxed criterion of 5% as the maximum 5mC level, since there were too few enhancers with absolutely 0% 5mC within a 1 kb window. Both 5mC+ and 5mC- groups had zero level for the 5hmC background. The mean 5mC levels in the 5mC+ and 5mC- group were 22 and 3%, respectively (*p-*value <1e-15). There were 1791 and 1365 putative enhancers in the 5mC + and 5mC- groups, respectively, which were all putative enhancers that met the above criteria. The distribution of H3K4me1 and H3K4me3 enrichments were estimated for each of the four groups (5hmC+, 5hmC-, 5mC+ and 5mC-) of enhancers.

### Graphical representation of 3-dimensional information

To better represent 3-dimensional genomics data, we developed R functions to produce scatter plots with automatic conversion of the third dimension to the color spectrum of data points. This substantially improved the insight into the data. These functions first eliminated outliers or incomplete data. We used the following criterion to remove data points as outliers to ensure at least 98% of the data are kept for the analysis. If the lower 1% and upper 99% quartiles were *Q*_1_ and *Q*_99_respectively, we defined *IQ* as the length of the interval between them: *IQ = Q*_*99*_ - *Q*_1_. A data point *x* is considered an outlier if either *x-Q*_*99*_ *>* 1.1*IQ*, or *Q*_*1*_ *-x >* 1.1*IQ,* i.e. *x* is outside the interval between *Q*_1_ and *Q*_99_ at a distance higher than 10% of such interval. The incomplete data are those lacking required CpGs in the window around a genomic site to infer the DNA methylation level. We then sorted all data points according to the third dimension into a particular number of bins (identified as function argument) to produce equal-width bins in the whole range of the 3rd dimension. Data points of each bin were subsequently assigned the same color of the whole spectrum. The data points were interpolated with a triangle-based linear method and projected them onto a 3-dimensional surface to ensure that the visual representation was not biased to the points overlaying other points. The scatter plots representing H3K4me1, H3K4me3 and DNA methylation in different cell types were produced by this method.

### Peak intersection analysis between WT and KO cells

Cross-normalization of processed ChIP-seq data was performed with the MAnorm software [77]. The same software was used to identify the common and specific peaks for pairs of cell types (WT versus KO). A peak was called specific to one cell type if the normalized enrichment value had more than a 2-fold change between the two cell types with a *p*-value <1e-5. Transcriptional activity on each peak was estimated by log_2_(1 + *m*), where *m* is the maximum number of RNA-seq reads aligned to the same genomic position inside the peak. Peaks specifically enriched of H3K4me3 and transcription were classified into 15 categories (Intron, LTR, Exon, Intergenic, 3’UTR, SINE, CpG island, Promoter, TSS, LINE, Simple repeat, 5’UTR, Non-coding, Low complexity, DNA Transposition) according to the genomic region using the HOMER suite. The small classes with less than 100 peaks were merged and labeled as an additional 16th category “Others”.

### R packages used in the analysis

We used the following R packages in our analysis: scales, intervals, modeest, bioDist, Hmisc, e1071, rpart, data.table, abind, plyr [85], raster, gplots, ggplot2 [86], pheatmap, reshape [87], multicore, zoo [88], directlabels, Biobase [89], GEOquery [90], limma [91].

## Additional file

Additional file 1: (PDF 162 kb)

## Abbreviations

5hmC: 5-hydroxymethylcytosine
5mC: 5-methylcytosine
ADD: ATRX-DNMT3-DNMT3L
Bis-seq: Bisulfite sequencing
*Btf3*: Basic transcription factor 3
*Cchcr1*: Coiled-coil α-helical rod protein 1
CFP1: CXXC finger protein 1
ChIP-seq: Chromatin Immunoprecipitation sequencing
ChIP-seq: Chromatin immunoprecipitation sequencing
COMPASS: COMplex of Proteins Associated with Set1
CpGMM: CpG methylation motif
DMT: DNA Methyl-Transferase
*Eif4g2*: Eukaryotic translation initiation factor 4, gamma 2
EMBL-EBI: European Molecular Biology Laboratory - European Bioinformatics Institute
ESC: Embryonic stem cell
FDR: False Discovery Rate
GEO: Gene Expression Omnibus
GO: Gene Ontology
*Gys2*: Glycogen synthase 2
H3: Histone 3
HMT: Histone Methyl-Transferase
IGV: Integrative Genomics Viewer
*Jade1*: Jade family PHD finger 1
Kb: Kilo base
KO: Gene knockout
*Ldhc*: Lactate dehydrogenase C
LINE: Long Interspersed Nuclear Element
LTR: Long Terminal Repeat
MBD: Methyl-CpG-Binding Domain
Med: Median DNA methylation
MEF: Mouse embryonic fibroblast
NCBI: National Center for Biotechnology Information
*P4hb*: Prolyl 4-hydroxylase subunit β
*Pawr*: Pro-apoptotic WT1 regulator
*Pkm*: Muscle pyruvate kinase
Pol2: RNA polymerase 2
*Ppp4c*: Protein phosphatase 4, catalytic subunit
RNA-seq: RNA sequencing
RRBS: Reduced Representation Bisulfite Sequencing
SINE: Short Interspersed Nuclear Element
TAB-seq: Tet-assisted bisulfite sequencing
*Tcf19*: Transcription factor 19
*Tet1*: The epigenetic regulators tet methylcytosine dioxygenase 1
TF: Transcription factor
TSS: Transcription Start Site
TTS: Transcription Termination Site
WCE: Whole cell extract
WGBS: Whole-Genome Bisulfite Sequencing
WT: Wild type
