# Supplementary material for: DNA methylation regulates discrimination of enhancers from promoters through a H3K4me1-H3K4me3 seesaw mechanism
Source: BMC Genomics. 2017 Dec 12;18:964. doi: 10.1186/s12864-017-4353-7 (PMC5727985; doi:10.1186/s12864-017-4353-7)
Supplement: Additional file 1: — (PDF 162 kb) [file 12864_2017_4353_MOESM1_ESM.pdf]

DNA methylation regulates discrimination of enhancers from promoters through a H3K4me1-H3K4me3 seesaw mechanism

Ali Sharifi-Zarchi[1,2,3,4*], Daniela Gerovska[5*], Kenjiro Adachi[6], Mehdi Totonchi[3], Hamid Pezeshk[7,8], Ryan J. Taft[9], Hans R. Schöler[6,10], Hamidreza Chitsaz[2], Mehdi Sadeghi[8,11], Hossein Baharvand[3,12#], Marcos J. Araúzo-Bravo[5,13,14#]

[1] Department of Bioinformatics, Institute of Biochemistry and Biophysics, University of Tehran, Tehran, Iran

[2] Computer Science Department, Colorado State University, Fort Collins, Colorado, USA

[3] Department of Stem Cells and Developmental Biology at Cell Science Research Center, Royan Institute for Stem Cell Biology and Technology, ACECR, Tehran, Iran

[4] Department of Computer Engineering, Sharif University of Technology, Tehran, Iran

[5] Computational Biology and Systems Biomedicine, Biodonostia Health Research Institute, 20014 San Sebastián, Spain

[6] Department of Cell and Developmental Biology, Max Planck Institute for Molecular Biomedicine, Münster, Germany

[7] School of Mathematics, Statistics and Computer Science, College of Science, University of Tehran, Tehran, Iran

[8] School of Biological Sciences, Institute for Research in Fundamental Sciences (IPM), Tehran, Iran

[9] Division of Genomics and Computational Biology, Institute for Molecular Bioscience, The University of Queensland, Brisbane, Australia

[10] Medical Faculty, University of Münster, Münster, Germany

[11] National Institute of Genetic Engineering and Biotechnology (NIGEB), Tehran, Iran

[12] Department of Developmental Biology, University of Science and Culture, ACECR, Tehran, Iran

[13] Computational Biology and Bioinformatics Group, Max Planck Institute for Molecular Biomedicine, Münster, Germany

[14] IKERBASQUE, Basque Foundation for Science, 48011 Bilbao, Spain

[*] These authors contributed equally

[#] Corresponding authors

E-mail: baharvand@royaninstitute.org (HB), mararabra@yahoo.co.uk (MJA-B)

Supplementary material captions

Fig. S1 Correlation of chromatin marks and gene transcription regulators with DNA methylation in distal putative enhancers. The distal putative enhancers (that are located outside gene coding regions, and have a distance of minimum 3 kb with the nearest TSS) are split into **(a)** DNA hypermethylated (DNA methylation > 0.5) and **(b)** DNA hypomethylated (DNA methylation ≤ 0.5) groups. In each DNA methylation group, distal enhancers are classified based on their genomic location (rows). For each class, Spearman's rank correlations between DNA methylation of ESCs and 9 different chromatin marks, the repressive histone 3 (H3), the gene transcription marker RNA polymerase 2 (Pol2), the enhancer marker histone acetyltransferase P300, and insulator marker CCCTC-binding factor are presented in columns. Red, white and blue colors show positive, null and negative correlations, respectively.

Fig. S2 Correlation of chromatin marks and gene transcription regulators with DNA methylation in 200 bp windows. The putative enhancers and promoter are split into **(a)** DNA hypermethylated (DNA methylation > 0.5) and **(b)** DNA hypomethylated (DNA methylation ≤ 0.5) groups. The DNA methylation and enrichment of chromatin marks are measured within a 200 bp window (+/- 100 bp) around the TSS or putative enhancer location.

Fig. S3 Correlation of chromatin marks and gene transcription regulators with DNA methylation in 400 bp windows. The putative enhancers and promoter are split into **(a)** DNA hypermethylated (DNA methylation > 0.5) and **(b)** DNA hypomethylated (DNA methylation ≤ 0.5) groups. The DNA methylation and enrichment of chromatin marks are measured within a 400 bp window (+/- 200 bp) around the TSS or putative enhancer location.

Fig S4. Proximal and distal H3K27me3 and H3K4me3 cell-type specific peaks in WT and *Dnmt1* KO MEF cells.

Figure S1

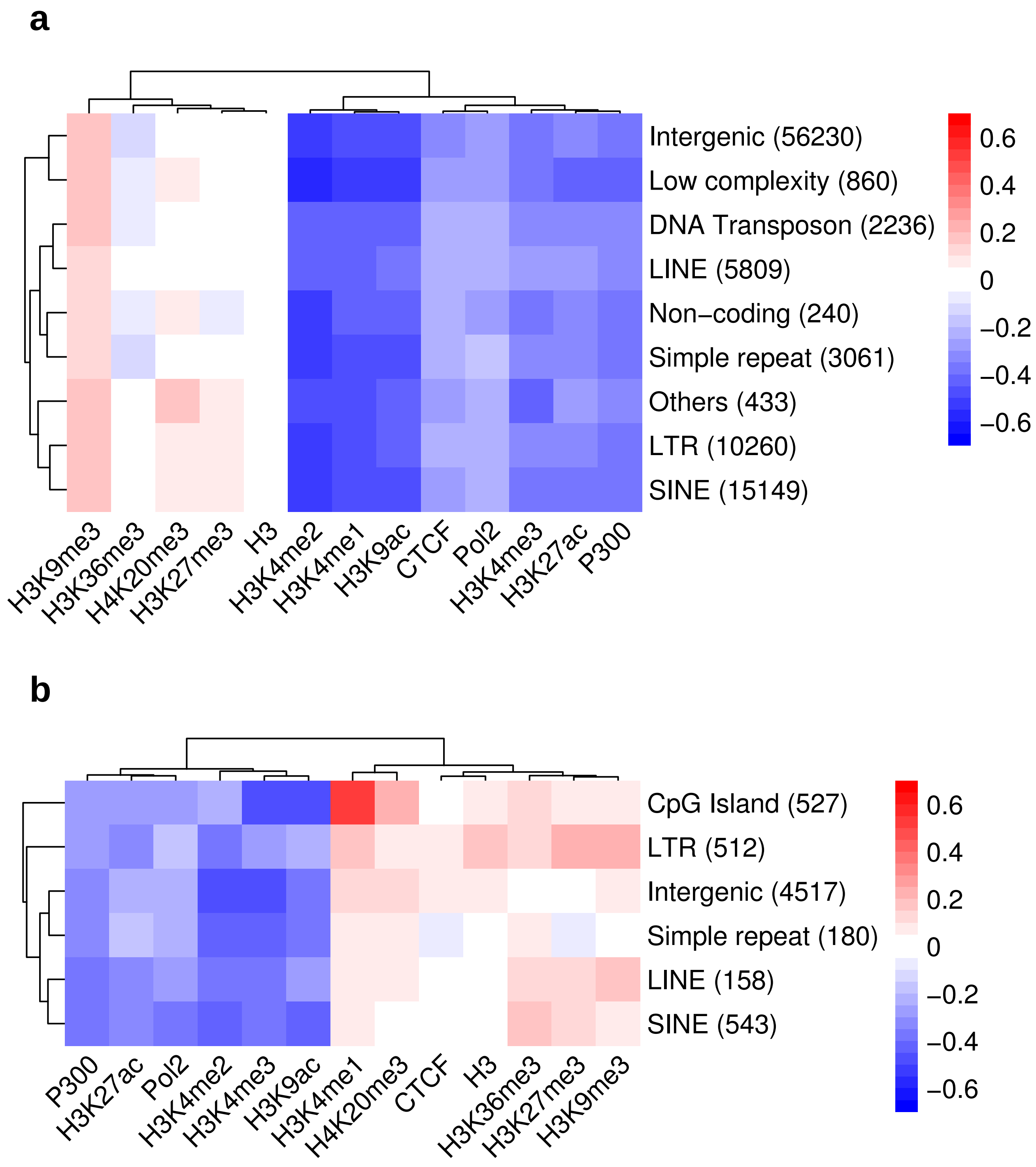
a
Intergenic (56230)
Low complexity (860)
DNA Transposon (2236)
LINE (5809)
Non−coding (240)
Simple repeat (3061)
Others (433)
LTR (10260)
SINE (15149)
H3K9me3
H3K36me3
H4K20me3
H3K27me3
H3
H3K4me2
H3K4me1
H3K9ac
CTCF
Pol2
H3K4me3
H3K27ac
P300
0.6
0.4
0.2
0
−0.2
−0.4
−0.6
b
CpG Island (527)
LTR (512)
Intergenic (4517)
Simple repeat (180)
LINE (158)
SINE (543)
P300
H3K27ac
Pol2
H3K4me2
H3K4me3
H3K9ac
H3K4me1
H4K20me3
CTCF
H3
H3K36me3
H3K27me3
H3K9me3
0.6
0.4
0.2
0
−0.2
−0.4
−0.6

Figure S2

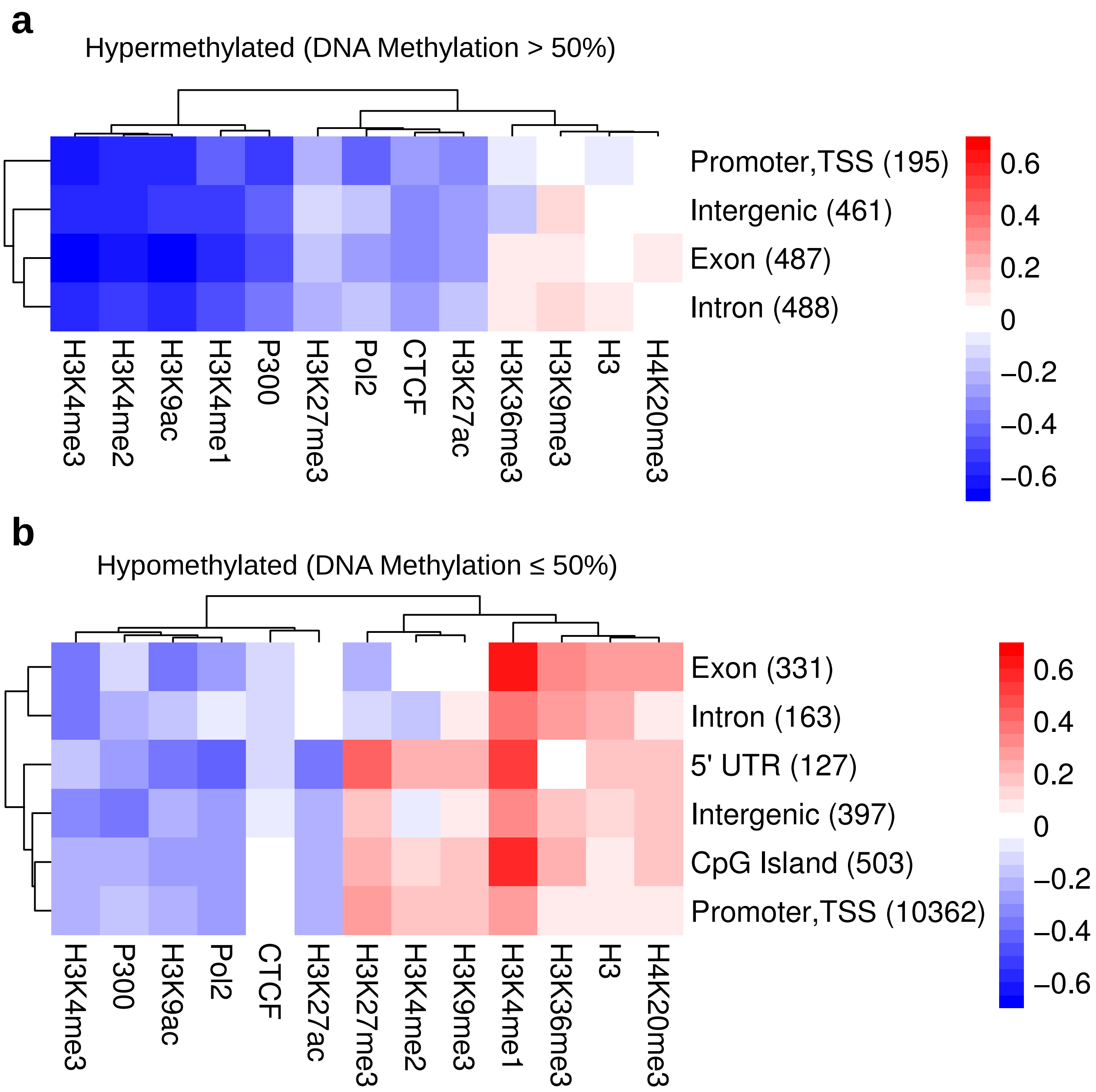
a
Hypermethylated (DNA Methylation > 50%)
Promoter,TSS (195)
Intergenic (461)
Exon (487)
Intron (488)
H3K4me3
H3K4me2
H3K9ac
H3K4me1
P300
H3K27me3
Pol2
CTCF
H3K27ac
H3K36me3
H3K9me3
H3
H4K20me3
0.6
0.4
0.2
0
−0.2
−0.4
−0.6
b
Hypomethylated (DNA Methylation ≤ 50%)
Exon (331)
Intron (163)
5' UTR (127)
Intergenic (397)
CpG Island (503)
Promoter,TSS (10362)
H3K4me3
P300
H3K9ac
Pol2
CTCF
H3K27ac
H3K27me3
H3K4me2
H3K9me3
H3K4me1
H3K36me3
H3
H4K20me3
0.6
0.4
0.2
0
−0.2
−0.4
−0.6

Figure S3

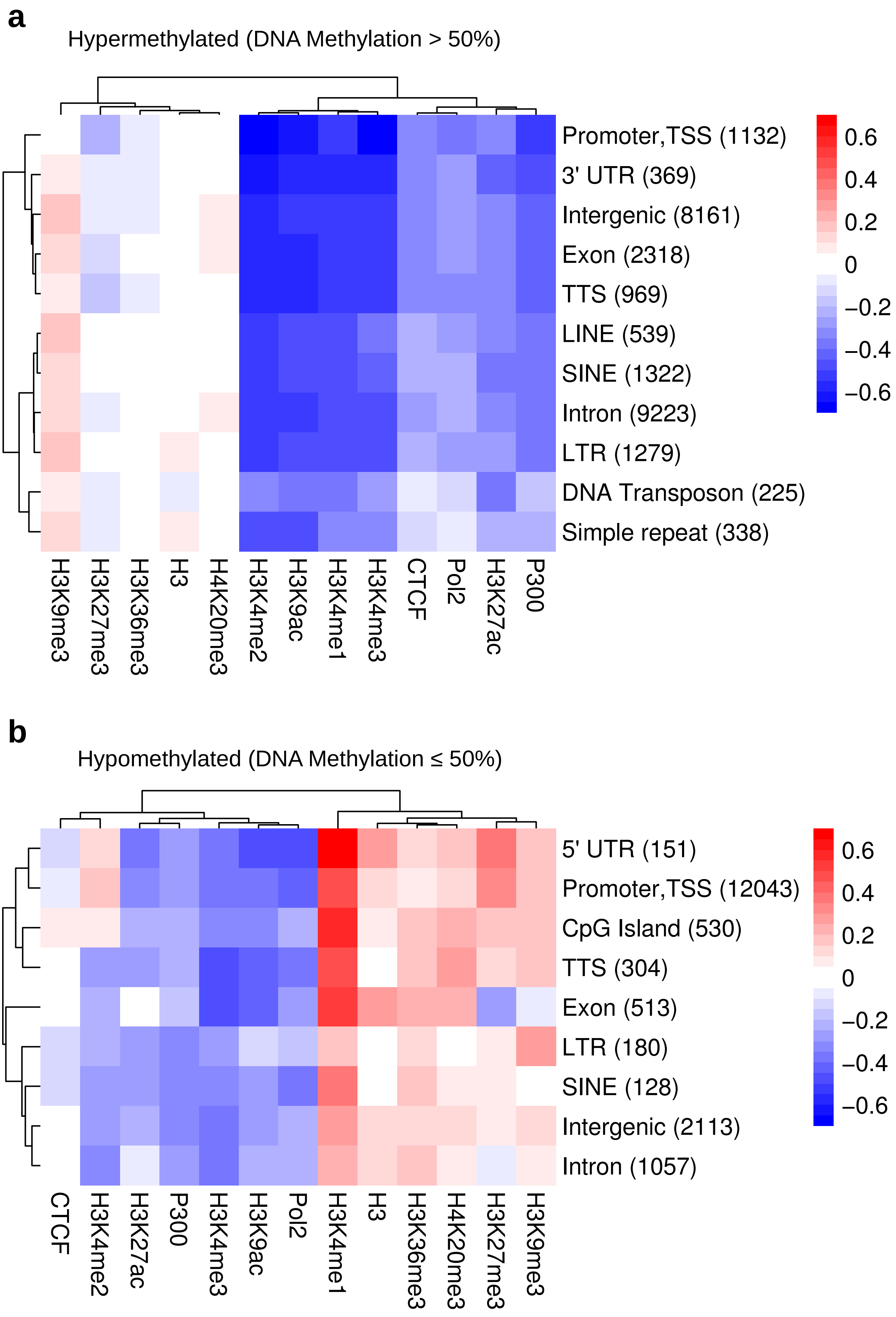
a
Hypermethylated (DNA Methylation > 50%)
Promoter,TSS (1132)
3' UTR (369)
Intergenic (8161)
Exon (2318)
TTS (969)
LINE (539)
SINE (1322)
Intron (9223)
LTR (1279)
DNA Transposon (225)
Simple repeat (338)
H3K9me3
H3K27me3
H3K36me3
H3
H4K20me3
H3K4me2
H3K9ac
H3K4me1
H3K4me3
CTCF
Pol2
H3K27ac
P300
0.6
0.4
0.2
0
−0.2
−0.4
−0.6
b
Hypomethylated (DNA Methylation ≤ 50%)
5' UTR (151)
Promoter,TSS (12043)
CpG Island (530)
TTS (304)
Exon (513)
LTR (180)
SINE (128)
Intergenic (2113)
Intron (1057)
CTCF
H3K4me2
H3K27ac
P300
H3K4me3
H3K9ac
Pol2
H3K4me1
H3
H3K36me3
H4K20me3
H3K27me3
H3K9me3
0.6
0.4
0.2
0
−0.2
−0.4
−0.6

Figure S4

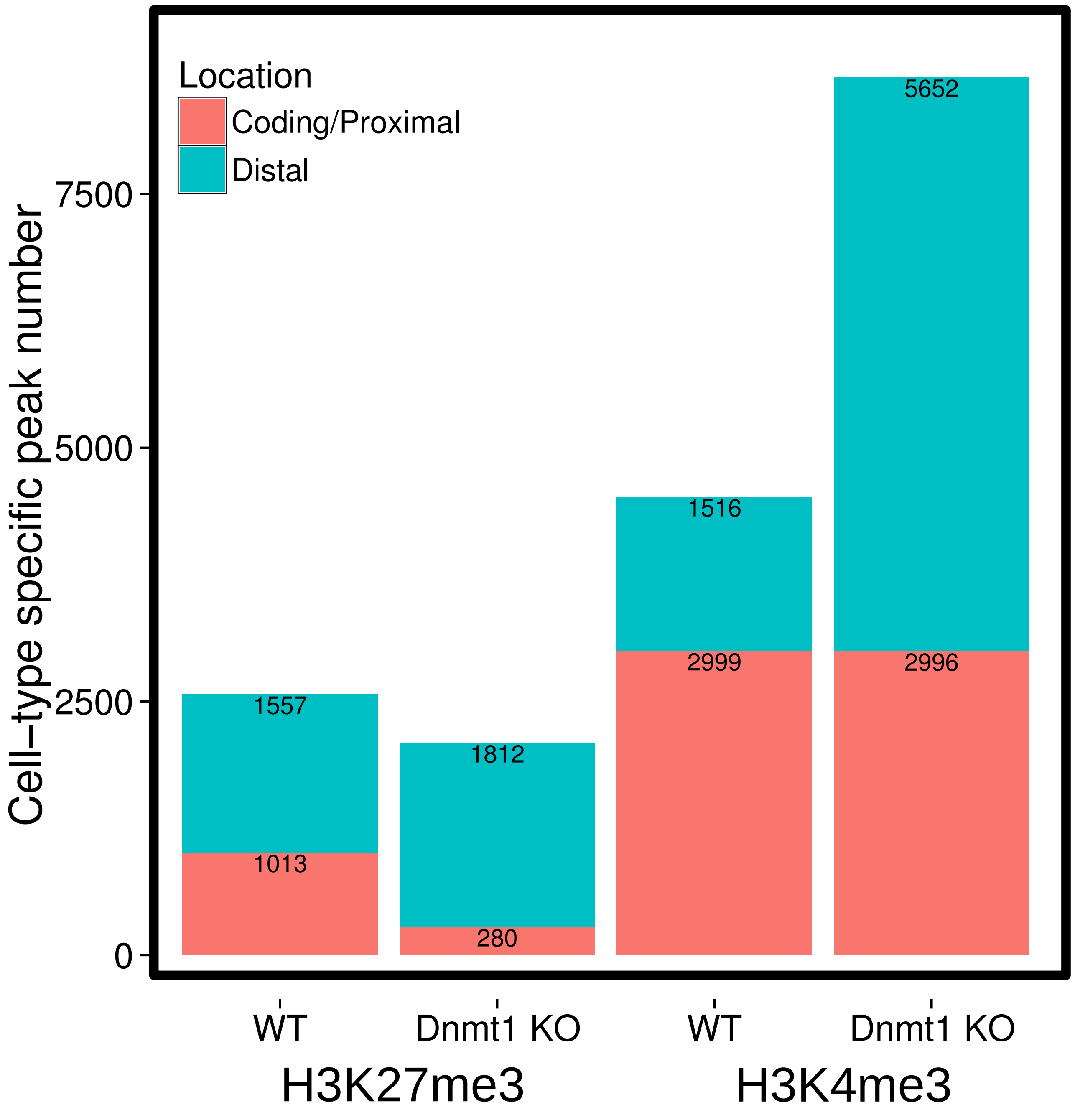

Location
Coding/Proximal
Distal
Cell–type specific peak number
7500
5000
2500
0
5652
1516
2999
2996
1557
1812
1013
280
WT
Dnmt1 KO
WT
Dnmt1 KO
H3K27me3
H3K4me3
